# Identification of potentially oncogenic alterations from tumor-only samples reveals Fanconi anemia pathway mutations in bladder carcinomas

**DOI:** 10.1038/s41525-017-0032-5

**Published:** 2017-10-03

**Authors:** Chioma J Madubata, Alireza Roshan-Ghias, Timothy Chu, Samuel Resnick, Junfei Zhao, Luis Arnes, Jiguang Wang, Raul Rabadan

**Affiliations:** 1 Department of Systems Biology, Columbia University, New York, NY 10032 USA; 2 Department of Biomedical Informatics, Columbia University, New York, NY 10032 USA; 3 Division of Life Science and Department of Chemical and Biological Engineering, Hong Kong University of Science and Technology, Kowloon, Hong Kong

## Abstract

Cancer is caused by germline and somatic mutations, which can share biological features such as amino acid change. However, integrated germline and somatic analysis remains uncommon. We present a framework that uses machine learning to learn features of recurrent somatic mutations to (1) predict somatic variants from tumor-only samples and (2) identify somatic-like germline variants for integrated analysis of tumor-normal DNA. Using data from 1769 patients from seven cancer types (bladder, glioblastoma, low-grade glioma, lung, melanoma, stomach, and pediatric glioma), we show that “somatic-like” germline variants are enriched for autosomal-dominant cancer-predisposition genes (*p* < 4.35 × 10^−15^), including *TP53*. Our framework identifies germline and somatic nonsense variants in *BRCA2* and other Fanconi anemia genes in 11% (11/100) of bladder cancer cases, suggesting a potential genetic predisposition in these patients. The bladder carcinoma patients with Fanconi anemia nonsense variants display a *BRCA*-deficiency somatic mutation signature, suggesting treatment targeted to DNA repair.

## Cancer: Analytic tool reveals inherited and non-inherited tumor-causing alterations

Bladder cancer cells often harbor DNA mutations that occur after tumor development, including some mutations that affect DNA repair. Raul Rabadan, Jiguang Wang, and colleagues from Columbia University in New York, USA, developed an analytic framework for identifying genetic variants, both inherited and newly arisen, that contribute to tumor development. The machine-learning tool—known as Tumor-Only Boosting Identification, or TOBI—learns what’s a cancer-associated mutation from a small training set of tumor samples and matched healthy controls. The researchers then fed the algorithm data from the tumors of 1769 patients with cancers of the bladder, brain, lungs, stomach and skin. They found that TOBI pinpointed many inherited and non-inherited mutations known to contribute to cancer growth. In bladder cancer samples, the tool also revealed a previously unknown role for inherited mutations in BRCA2 and other DNA repair genes in the so-called Fanconi anemia pathway.

## Introduction

Cancer often results from specific DNA alterations, and identification of cancer-causing mutations underlies genome-based precision cancer treatment.^1^ Somatic mutations can be identified by sequencing matched tumor and normal DNA,^2^ where normal samples can come from blood or any other non-tumor tissue, and then removing any shared variants (germline variants). This paired tumor-normal analysis has identified oncogenic somatic mutations in multiple cancer types, including cohorts originally analyzed by The Cancer Genome Atlas (TCGA).^3–6^

Despite the value in sequencing matched normal DNA to truly differentiate germline and somatic variants,^7^ the historically high cost of sequencing led to tumor-only sequencing in many research projects^8–10^ and clinical settings.^11^ Tumor-only sequences contain both germline and somatic alterations, but differentiating the 10–100s of somatic mutations^12–14^ from tens of thousands of germline variants remains challenging. Common attempts to identify somatic variants from tumor-only WES data involve removing dbSNP^15^ mutations common in the general population and focusing on genes in the Catalogue Of Somatic Mutations In Cancer (COSMIC).^16^ These strategies fail to recognize private polymorphisms that are not annotated in public repositories and preclude the discovery of novel oncogenic events.

A limited number of computational strategies exist to identify somatic variants from tumor-only WES data. Certain strategies rely on a single patient’s sequence alignment information, either predicting somatic deletions based on read-pair alignments and read depth^9^ or predicting somatic single nucleotide variants (SNV) using base quality, variant allele frequency (VAF), and sequencing error.^17^ Other strategies use population allele frequency tabulated from a cohort of normal genomes to remove potential germline SNPs.^18^ None of these techniques integrate information from both the individual patient sequence and the total patient cohort. These techniques also fail to leverage valuable databases of somatic mutations or predicted mutation effects.

Integrated information from individual patients, patient cohorts, and databases can inform an alternative approach that learns biological features from known somatic variants in order to predict somatic variants from tumor-only samples. This approach would require a patient cohort with some matched tumor-normal cases and some tumor-only cases. The tumor-normal cases would form a test set for identifying true somatic mutations, and the biological features of these confirmed somatic variants would be used to classify variants from the remaining tumor-only samples. Prior studies of mixed tumor-normal and tumor-only cohorts used manual recurrence analysis of specific genes to reveal altered genes in lymphoma,^19, 20^ relapsed pediatric acute lymphoblastic leukemia,^21^ and pediatric glioma,^22^ but the focus on gene identity had decreased power to identify oncogenic variants. In contrast, the approach we suggest would use machine learning instead of manual analysis, make predictions across the whole exome instead of focusing on specific genes, and use multiple biological features to increase power to predict somatic variants.

While tumor-only analysis remains common and somatic mutations associate with cancer development, germline DNA alterations can also be oncogenic.^2^ A standardized framework for unified analysis of germline and somatic variants could reveal key oncogenic pathways. Recent analysis of sporadic ovarian cancer found significantly enriched germline and somatic alterations in the Fanconi anemia (FA) and MAPK pathways.^23^ Furthermore, certain oncogenic germline variants share biological features with known somatic variants, such as affecting the same amino acid.^24^ A machine learning framework built upon biological features of somatic variants would have high power to identify germline variants with somatic features that might influence tumor development.

Thus, we present a framework to address the separate but related challenges of tumor-only somatic analysis and integrated germline–somatic analysis. Our Tumor-Only Boosting Identification framework (TOBI) learns from a small training set of tumor-normal pairs to generate a classification model that identifies variants with somatic characteristics from tumor-only samples. If normal DNA is available, we can assess whether TOBI predicted certain germline variants as somatic; we refer to these variants as “somatic-like” germline variants. Somatic-like germline variants complement the somatic landscape, promoting integrated analysis of oncogenic processes. TOBI uses gradient boosting, a machine learning algorithm with consistently superior performance in diverse classification tasks.^25^ Using 1769 patients across seven tumor types, we developed TOBI, evaluated TOBI’s ability to identify somatic variants, and identified somatic-like germline variants (SLG variants), including variants with known or possible oncogenic potential.

## Results

### Framework for predicting somatic, germline and SLG variants

**Fig. 1:**
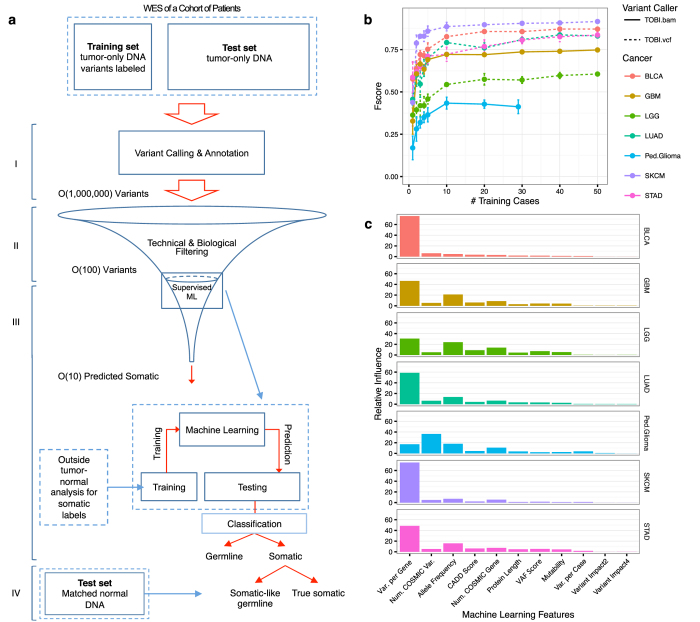
TOBI framework and features. **a** Outline for predicting somatic variants with TOBI. TOBI accepts tumor-only DNA, separated into a training set of cases with prior tumor-normal somatic analysis available and a test set. The steps of TOBI analysis are (I) variant calling and annotation, (II) filtering, (III) machine learning to classify “somatic” and “germline” variants, and (IV) identification of somatic-like germline variants. Step III predictions result in tens of predicted somatic variants per case. **b** Average *F*-score for increasing numbers of cases in the training set in seven cancer types. Number of samples in the training set equals number in testing set. Points represent average predictions from five runs with randomly selected training and testing sets cases; error bars represent +/− s.e.m. TOBI.bam indicates samples were analyzed from aligned sequence files (.bam) using TOBI steps I-III; TOBI.vcf indicates samples were analyzed from variant call files (.vcf) using TOBI steps II–III. **c** Relative importance of features in gradient boosting classification model generated from a training set with twenty cases in each individual cancer

Our framework consists of four main steps: steps I-III accommodate tumor WES data at different stages of analysis, and step IV incorporates germline VAF when available (Fig. 1a). Step I receives aligned WES files (.bam files), calls variants against a human reference genome, and annotates variants (full details in Methods). These variant calls (.vcf files) are the input for Step II, allowing users to jump to Step II if they have previous annotated variants from tumor-only samples. Step II filters variants using biological and technical criteria described in the Methods, retaining high quality variants that are rare in the population (population minor allele frequency less than 1% in the 1000 Genomes Project^26^).

Step III receives the remaining training set variants and uses the gradient boosting machine learning algorithm to generate the somatic classification model. Gradient boosting generates a classifier from an ensemble of decision trees, where each subsequent tree learns from the previously misclassified training set observations.^27^ For example, some features of previously described highly-recurrent variants will easily classify hotspot variants, while other features will be more relevant for classifying rarer mutations in subsequent trees. We optimized the gradient boosting parameters using systematic grid search (Methods). Each variant in the training set represents an observation for machine learning. Ten biological features were used for gradient boosting (full features in Supplementary Text); features include database-derived features from COSMIC, cohort-associated features such as “Variants per Gene”, and individual sequence features such as tumor VAF. Model generation requires training set variants annotated with true somatic status, defined by a user-generated list of somatic variants output from separate somatic variant calling pipelines (e.g., MuTect,^28^ SAVI^29^). Step III ends by applying the final somatic classification model to the test set variants.

Finally, Step IV occurs only if normal WES DNA is available for test set samples, and distinguishes somatic variants from somatic-like germline variants.

### TOBI training and test sets

We developed TOBI using glioblastoma multiforme (GBM) cases from TCGA,^3^ and assessed TOBI on five adult cancer types from TCGA: bladder urothelial carcinoma (BLCA),^6^ brain lower grade glioma (LGG),^4^ lung adenocarcinoma (LUAD),^5^ skin cutaneous melanoma (SKCM),^30^ and stomach adenocarcinoma (STAD).^31^ We used TCGA’s previously published somatic calls as the “true somatic” calls for labeling training set variants. To assess TOBI’s performance on pediatric tumors, we analyzed pediatric glioma cases (Ped.Glioma), including cases with published tumor-normal analysis^10, 32^ and tumor-only cases.^8, 10, 22^ The number of cases per cancer type, and the number of cases used in each figure, is in Supplementary Table 1a.

Since cancer-sequencing studies have variable numbers of paired tumor-normal samples,^8–10^ we assessed the number of training cases required for model generation (Fig. 1b). Increasing the number of training set tumor samples from 1 to 50 samples improved performance, with *F*-scores plateauing between 20 and 50 training cases in the six adult cancers. Twenty training cases produced an average *F*-score within 10% of the *F*-score at the maximum training set size (Supplementary Table 1b). Thus, in the remainder of our analysis, we used 20 random cases as the training set size and all remaining cases as the test set to reflect a WES scenario where the majority of patient samples are tumor-only.

Historical tumor-only samples may be formalin-fixed and paraffin-embedded (FFPE), which introduces sequencing artifacts. We applied TOBI’s LUAD classification model to FFPE LUAD cases (Supplementary Fig. 7, Supplementary Table 7), and observed a slightly decreased *F*-score for FPPE (0.68) vs. frozen samples (0.81). FFPE samples had similar sensitivity and specificity (0.94, 0.97) compared to frozen samples (0.87, 0.96).

Next, we assessed how differences in patient ancestry, sequencing institution, or hypermutator status within a cohort might affect TOBI performance. Stratifying on a patient’s reported race, TOBI had decreased mean *F*-scores when the training and test set differed by race in almost all cancers (Supplementary Fig. 2, Supplementary Table 2c). Differing sequencing institutions between the training and test set also generated lower mean *F*-scores in almost all cross-institutional predictions (TCGA GBM with a cohort of 80 additional non-TCGA cases^33^ in Supplementary Fig. 3a–c and Supplementary Table 3; Ped.Glioma analysis in Supplementary Fig. 3d). Finally, using hypermutator status from the STAD publication,^31^ we found no significant effect on TOBI’s performance when analyzing a non-hypermutator population or mixed population (61 hypermutator, 219 non-hypermutator) (Supplementary Fig. 4, Supplementary Table 4). Thus, TOBI’s performance might improve with features denoting patient race or institutional differences, but performance appears robust to hypermutator samples.

### TOBI features

We assessed the importance of our ten biological features to a cancer type’s final classification model using relative influence,^34^ a measure of how frequently one feature is used in the decision trees within the final classification model (Fig. 1c). In all adult cancers, the feature with greatest relative influence was “Variants in Gene”, the total number of variants per gene normalized by cohort size. In pediatric glioma, the feature with greatest relative influence was “Num. COSMIC Var.”, representing the number of cases in COSMIC with a specific variant; this may reflect both the lower mutation burden in pediatric glioma and the prevalence of hotspot mutations in *H3F3A*. As expected, removal of these top features from the classification model caused a slight drop in *F*-score, while removal of other individual features or both COSMIC-derived features minimally affected performance (Supplementary Fig. 1).

### High performance somatic variant identification

**Fig. 2:**
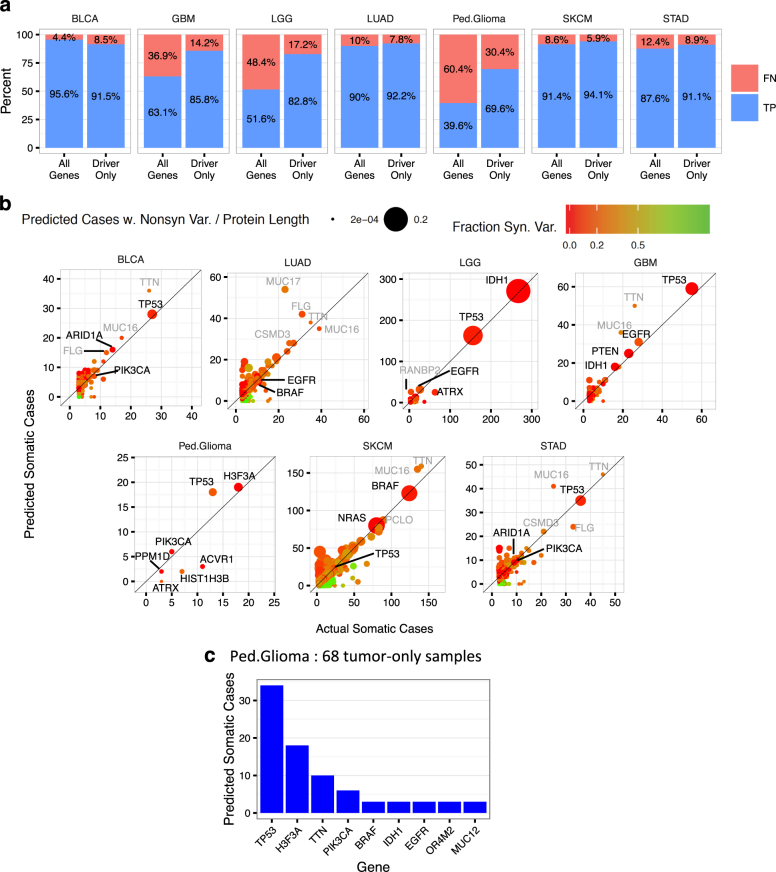
Somatic variant prediction captures driver genes. **a** For each indicated cancer type, top panel shows percentages of true positive (TP) or false negative (FN) TOBI somatic predictions in nonsynonymous variants across all genes or only driver genes. **b** Comparison of actual vs. predicted cases with somatic, nonsynonymous variants in each cancer type. Dot color corresponds to the fraction of synonymous variants out of all variants remaining after TOBI filtering (Step II); dot size corresponds to number of predicted cases over protein length in amino acids. Driver genes labeled in black; other genes in the top five most predicted cases labeled in gray. For clarity, genes with less than three previously published somatic variants are not shown. **c** Number of cases with predicted somatic variants when pediatric glioma classification model is applied to 68 tumor-only samples; genes predicted in at least three cases shown. For all cancers, twenty randomly selected tumor-normal cases comprised training set; remaining paired tumor-normal samples formed testing set

We compared TOBI’s somatic classifications to published somatic calls from tumor-normal analysis of test set cases,^3–6, 30, 31, 10, 32^ Across all variants, TOBI had a sensitivity of 86.6%; for nonsynonymous variants, TOBI had a sensitivity of 87.2%. Additional performance metrics are in Supplementary Table 5 and Supplementary figure 5a. TOBI also has high sensitivity for variants with tumor VAF as low as 5% (Supplementary figure 5b,c). Per gene, the number of cases with nonsynonymous variants predicted as somatic closely matches published somatic analysis (Figs. 2a, b). TOBI’s sensitivity in a cancer type positively correlates with the median somatic SNV per megabase (Mb) across all cases of that cancer (Spearman rho 0.964, *p*-value < 0.003 for both all gene and driver only sensitivity, Supplementary Figure 13). TOBI predictions on previously published somatic mutations are in Supplementary Table 6.

While TOBI identifies variants with somatic characteristics, an important challenge in precision medicine involves finding genes that promote tumor development (“driver genes”). Thus, we assessed whether TOBI’s predictions were enriched for driver genes in each tumor type, defining driver genes as those with evidence of positive selection in somatic mutation patterns as published by the Intogen group.^35^ In six cancers, TOBI has a higher true positive rate of nonsynonymous variants in driver genes compared to all genes (Fig. 2b). Such enrichment occurred despite training sets retaining synonymous variants and probable passenger variants. This driver gene enrichment did not solely arise from predicting highly recurrent genes, as suggested by TOBI’s similar performance in high, medium, and low recurrence genes in most cancers (Supplementary Fig. 6).

Finally, to demonstrate analysis of a truly tumor-only data set, we applied the pediatric glioma classification model to 68 tumor-only cases (Fig. 2c), identifying known driver genes in pediatric glioma (*TP53*, *H3F3A*, *PIK3CA*). All predicted *BRAF* and *IDH1* variants occurred at known somatic hotspots (*BRAF* V600E, *IDH1* R132H).

### TOBI outperforms other tumor-only analysis tools

Using six GBM and six Ped.Glioma cases, we compared TOBI’s results to those from other software for tumor-only WES somatic variant analysis: Virtual Normal Correction (VNC)^18^ and SomVarIUS^17^ (Supplementary Table 8). Compared to VNC, TOBI has higher *F*-scores (0.48 for Ped.Glioma and 0.22 for GBM; VNC *F-*score less than 0.0002 for both Ped.Glioma and GBM). SomVarIUS did not identify any true somatic mutations in Ped.Glioma. TOBI also predicts orders of magnitude fewer somatic variants per case compared to VNC and SomVarIUS (TOBI: ~5–50; VNC: ~300,000; SomVarIUS: ~100–3000). TOBI’s higher *F*-scores and biologically appropriate number of somatic variants indicates that TOBI outperforms these methods.

We also compared TOBI to methods that assess a variant’s disease potential^36–39^ since these methods have been used to assess effects of somatic variants. Using published somatic variants from tumor-normal analysis as the gold standard, TOBI consistently had the highest AUC (Supplementary figure 8).

### Identification of “somatic-like” germline variants

Having established TOBI’s ability to identify somatic variants from tumor-only samples, we next assessed whether TOBI was capturing germline variants with somatic features. TOBI’s false positive (FP) variants could include germline variants that share features with true somatic variants, making them “somatic-like” germline (SLG) variants. SLG variants could be benign or oncogenic. Alternatively, FP variants might be tumor-specific variants that were not previously published due to variability in somatic variant analysis.^40^

First, we assessed TOBI’s overall false positive rate (FPR) in the cancer test sets. Since FP variants may include SLG variants, we also calculated the FPR from applying the Ped.Glioma classification model to a set of 100 non-tumor exomes from individuals without cancer sequenced by the 1000 Genomes Project.^26^ The FPR in these 1000 Genomes individuals (median FPR 0.25%, range 0.15–1.62%) was significantly lower than the FPR in any of the cancer cohorts (Supplementary Fig. 9). The higher FPR from tumor cohorts suggests that some FP calls represent somatic-like germline variants.

**Fig. 3:**
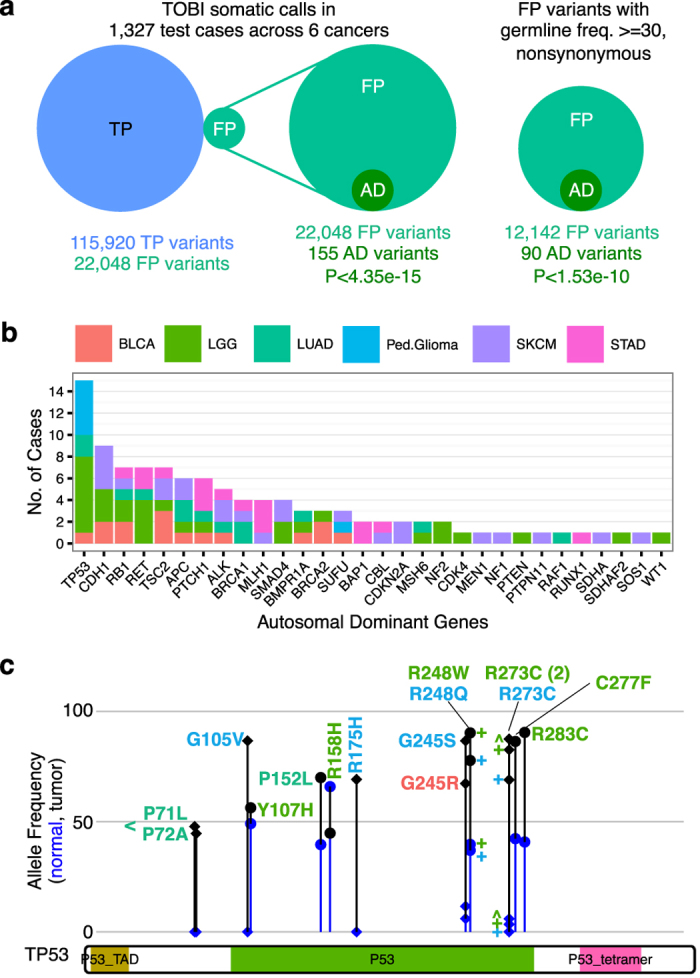
“Somatic-like” germline (SLG) variants are enriched for genes associated with autosomal dominant cancer-predisposition syndromes (AD genes). **a** Variants predicted as somatic by TOBI include 22,048 variants not reported as somatic in published analysis of 1327 cases from five adult cancer types and pediatric glioma, with significant enrichment for AD genes in all FP variants and the subset of nonsynonymous variants with germline allele frequency greater than 30%. *p*-value from Poisson cumulative distribution. **b** Distribution of patient cases with FP variants in AD genes. Cancer abbreviations and color consistent with Figs. 1 and 2. **c** FP variants in *TP53* domains. Height of line represents allele frequency, with normal frequency at the blue point and tumor frequency in black. Circles indicate patients where normal frequency of variant is greater than or equal to 30%; diamonds indicate normal frequency less than 30%. Color of variant name corresponds to cancer color in **b**. “<” indicates P71L and P72A occurred in same LUAD patient. “R273C (2)” indicates two patients with LGG had this variant. Colored “+” or “^” indicate individual patient allele frequencies

To identify SLG variants, we analyzed germline VAF from 1327 test cases in six cancers excluding GBM. VAF is the fraction of exome sequencing reads corresponding to the variant allele at a genomic site within a specific patient sample. To be classified as an SLG variant, a FP variant needed a germline VAF of at least 30% to decrease the probability that the germline variant represented tumor contamination or artifacts.^24^ Since certain germline variants highly increase predisposition to cancer,^24, 41^ we analyzed SLG variants for enrichment in 60 genes associated with autosomal dominant cancer-predisposition syndromes,^24^ or “AD genes” (listed in Supplementary Table 9), and found significant enrichment of AD genes in nonsynonymous SLG variants (*p* < 1.53 × 10^−10^; Fig. 3a). SLG nonsynonymous variants in *TP53* occurred in seven cases (Fig. 3c). Certain inactivating mutations in tumor suppressors are heterozygous germline variants, but show loss of heterozygosity in the tumor.^42^ Five of TP53 SLG variants exhibit evidence of loss of heterozygosity, with germline VAFs below 45% and tumor VAFs above 70%.

Focusing on nonsynonymous FP variants in AD genes, we found 15 cases with *TP53* mutations and at least seven cases with mutations in *CDH1*, *RB1*, *RET* or *TSC2* (Fig. 3b). In three Ped.Glioma cases, TOBI predicted somatic *TP53* variants with tumor VAF greater than 65% and germline VAF of 0% (Fig. 3c; variants G105V, R175H, and R273C). Despite the high tumor VAF and low germline VAF, these variants were not published as somatic variants in outside tumor-normal analysis,^10^ illustrating that TOBI can identify somatic variants that may be inconsistently called.

**Fig. 4:**
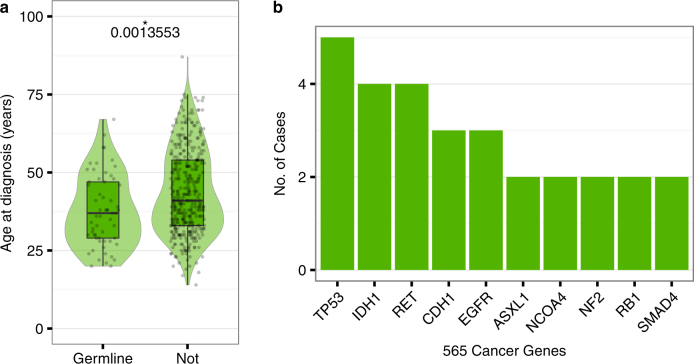
SLG variants in low-grade glioma associated with earlier age of diagnosis. **a** Distribution of diagnosis age in 492 LGG test set cases with or without nonsynonymous SLG variants in 565 cancer genes. For the violin plots, width of shape indicates density. In overlaid boxplots, the horizontal center line indicates the median (37 years vs. 41 years), upper and lower box edges correspond to the 25th and 75th percentiles, and the upper and lower whiskers extends from the closest box edge to the highest or lowest value within 1.5x the interquartile range, respectively. *p*-value calculated with two-sided Wilcoxon–Mann–Whitney test; * indicates *p* < 0.01. **b** Cancer genes with recurrent nonsynonymous SLG in LGG

Certain germline variants in cancer-associated genes correlate with earlier age of diagnosis,^41^ so we analyzed whether presence of nonsynonymous SLG variants in 565 cancer-associated genes^24^ (list in Supplementary Table 9) associated with earlier age of diagnosis in any cancer type. Supplementary table 10 provides the number of cases with SLG variants in these cancer-associated genes for each cancer type. In LGG, patients with cancer-associated SLG variants had significantly earlier age at diagnosis (median 37 years vs. 41 years, *p* = 0.0013; Fig. 4a; Supplementary Fig. 10). The most LGG cases had SLG variants in *TP53 (n = 4)*, followed by *IDH1* (three cases: V71I [COSM96923], one case: R82K [COSM4169909]) and *RET* (Y791F [COSM1159820], I852M [COSM4573611], R982H [COSM1264016], T1038A [COSM4650197]). Many genes with SLG variants in LGG have also shown recurrent somatic mutations in prior analysis^4^ (e.g., *TP53, IDH1*, *EGFR*, and *NF2*; Fig. 4b).

### Bladder cancer cases with inactivating mutations in FA pathway display somatic signature of *BRCA*-deficiency

Truncating germline alterations in cancer predisposition genes have been reported in 4–19% of cancer types.^41^ Accordingly, we examined the exome-wide SLG nonsense variants in each cancer type. Bladder carcinoma cases showed significant enrichment of SLG nonsense variants in the FA pathway based on pathway assessment with g:Profiler^43^ (49 genes with SLG variants, 54 genes in FA pathway, 3 overlapping genes; *p*-value of 0.029 after multiple testing correction; Supplementary Fig. 11). The FA pathway normally performs DNA repair of interstrand crosslinks, which requires homologous recombination.^44^

**Fig. 5:**
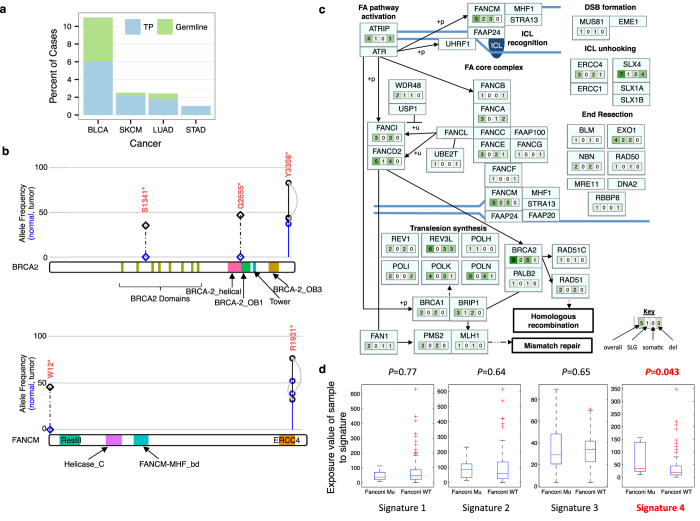
Enrichment for *BRCA*-deficiency somatic signature in bladder cancer patients with inactivating mutations in the Fanconi anemia (FA) pathway. **a** Percentage of test set cases with TOBI-somatic nonsense mutations; “Germline” indicates variant allele frequency (VAF) > = 30% in normal; “TP”, or true positives, were previously reported as somatic and have VAF < 30% in normal. Total number of test cases: 100 BLCA, 317 SKCM, 165 LUAD, and 199 STAD. **b** TOBI-somatic nonsense variants in *BRCA2* and *FANCM*; diamond and dashed line indicate TP variant; solid line and circle are germline; gray arrows go from VAF in normal to tumor. **c** FA pathway with number of altered cases in bladder cancer shown for each component. **d** Enrichment for signature 4 in BLCA FA nonsense mutant vs. wildtype samples. *p*-value calculated with rank sum test. *Mu* mutant, *WT* wildtype

We then assessed the overall occurrence of germline and somatic nonsense mutations in the FA pathway predicted by TOBI (Fig. 5a). In bladder cancer, TOBI predicted these variants in 11% (11/100) of patients. Less than 2.5% of patients in any other cancer type had predicted nonsense FA variants. True somatic nonsense variants occurred in 6% of BLCA cases, affecting genes *BRCA2*, *FANCM*, *FANCE*, *REV3L*, and *SLX4*. Germline nonsense variants were predicted in 5% of BLCA cases, affecting *BRCA2*, *FANCM*, and *FANCD2*. Several of these germline variants showed potential loss of heterozygosity based on increased VAF in tumor DNA compared to germline DNA (Fig. 5b: *FANCM* R1931*, *BRCA2* Y3308*). Of note, *BRCA2* variant Y3308* has been associated with hereditary colorectal and breast cancer.^45^ Mice ES cells with *BRCA2* Y3308* mutations showed hypersensitivity to ionizing radiation and crosslinking agents, as well as decreased homologous recombination efficiency.^46^ Additionally, FANCM R1931* was associated with increased breast cancer risk and deficient DNA repair.^47^ Fig. 5c and Supplementary Table 11 describe published somatic copy number alterations and predicted nonsynonymous variants within the FA pathway for this BLCA cohort.

Finally, we assessed whether BLCA cases with predicted FA pathway nonsense mutations had significantly different mutational signatures compared to wildtype cases. Using all somatic mutations published for 130 TCGA BLCA cases^6^ including our 100 test cases, we generated trinucleotide mutational spectra that decomposed into four somatic signatures (Supplementary Fig. 12a,b). Cases with FA nonsense mutations were only enriched in the fourth signature (Fig. 4c), a somatic signature similar to the BRCA1/2-deficiency signature from a pan-cancer analysis (signature 3 in the referenced publication).^48^ Enrichment of this somatic mutation signature in bladder cancer cases with nonsense FA variants suggests that these FA nonsense variants, whether somatic or germline, affect the bladder cancer somatic mutation landscape.

## Discussion

In this report, we present TOBI, a new unifying framework that uses the gradient boosting machine learning algorithm to identify somatic variants from tumor-only data or identify somatic-like germline variants in patients with tumor-normal DNA available. Our framework is available online for non-commercial use (https://github.com/RabadanLab/TOBI).

In tumor-only analysis, TOBI successfully identified 87% of nonsynonymous somatic variants. Higher true positive rates in driver genes suggest that TOBI enriches for cancer-causing variants. TOBI’s similar performance on frozen and FFPE samples suggests that TOBI filters certain FFPE artifacts. A TOBI modification trained on FFPE artifacts could potentially remove more FFPE sequencing artifacts, although this modification would need testing. TOBI also outperforms other methods designed for somatic variant identification from tumor-only samples. This higher performance likely reflects two fundamental differences between alternative methods and TOBI. First, alternative techniques use a single information source, but TOBI integrates biological features from individual variants, patient cohorts, and curated databases. Second, TOBI uses the powerful gradient boosting algorithm to classify variants, allowing TOBI to learn features important to specific tumor types (Fig. 1c).

When germline VAF information is available, TOBI can identify “somatic-like” germline variants. These SLG variants include oncogenic germline variants validated by outside groups, such as the TP53 R248Q alteration confirmed as germline by tumor-normal analysis of a pediatric glioma case.^10^ SLG variants in cancer genes also associated with earlier age of diagnosis in patients with low-grade glioma (Fig. 4a), suggesting that TOBI’s SLG variants are enriched for cancer-associated variants.

Analysis of bladder carcinoma cases using TOBI revealed largely unreported germline inactivating mutations in the FA pathway, suggesting a potential genetic predisposition in 5% of patients. Outside analysis of a 14-patient bladder tumor cohort^49^ found a germline nonsense variant in *BRCA2*, but did not assess FA mutations. Germline *BRCA2* nonsense mutations in bladder carcinoma may reflect the pan-cancer susceptibility attributed to germline *BRCA2* mutations in analysis of other adult cancers.^41^ Future assessment of a larger BLCA cohort may reveal associations between germline FA mutations and clinical outcomes, similar to how an expanded cohort of prostate cancer patients revealed significantly more deleterious germline mutations in DNA repair genes in patients with metastatic vs. localized prostate cancer.^50^

Our integrated somatic and germline analysis identified nonsense FA pathway mutations in 11% of BLCA cases, suggesting a role for aberrant interstrand crosslink repair in bladder tumor development. Enrichment for a *BRCA*-deficiency somatic signature in these patients indicates similarity between FA mutant bladder cancers and *BRCA*-mutant breast cancers. However, further biological experiments would clarify the role of the FA mutations in bladder cancer. Treating *BRCA*-mutant breast cancers with PARP inhibitors improved patient outcome,^51^ so PARP inhibitors may also show increased effectiveness in bladder tumors with *BRCA2* or other FA mutations. Additionally, recent research found that the presence of tumor DNA alterations in *FANCC* (a member of the FA pathway), *ATM*, and *RB1* predicted beneficial response to cisplatin neoadjuvant chemotherapy.^52^ Future research could determine whether FA nonsense mutations also predict beneficial response to Cisplatin, particularly given the beneficial response to cisplatin in patients with *BRCA1* mutant breast cancers.^53^

We recognize several limitations for the TOBI framework. First, TOBI’s biological features include some that depend on outside databases (COSMIC variants), and future versions of these databases could affect TOBI predictions. Moreover, we only assessed a subset of biological features; alternative features could lead to improved TOBI performance. Second, FFPE status, patient ancestry, and sequencing institution do affect TOBI’s performance, suggesting that TOBI will perform best on relatively homogeneous cancer cohorts. Third, TOBI’s sensitivity positively correlates with the median somatic SNV rate per cancers, possibly due to the increased fraction of somatic mutations in the training set of melanoma and other cancers with high mutation rates. This suggests that TOBI will be most sensitive in cancers with high somatic mutation rates. Fourth, for germline variant analysis, TOBI’s designation of SLG variants denotes “somatic-like” status, but does not differentiate oncogenic and benign germline variants. Finally, fully understanding the role of FA variants in bladder cancer requires experimental validation.

In sum, we propose a framework that analyzes either tumor-only samples or samples with matched tumor-normal DNA for variants with somatic features. In tumor-only samples, the framework (1) promotes the study of previously collected tumor samples without matched normal DNA, unlocking a vast repository of tumor-only samples without sequencing of matched normal DNA, and (2) prioritizes exome alterations in a particular patient by focusing on variants with somatic characteristics. In cases with matched normal DNA, this framework identifies germline variants that present somatic-like features and may inform tumor developments. Integrated analysis of germline and somatic variants remains uncommon, making TOBI’s identification of both somatic-like germline variants and somatic variants a unique strength. Applying the TOBI framework to seven cancer types illustrated that TOBI recovers known oncogenic variants of somatic and germline origin, and suggests a previously unreported role for inactivating mutations in the FA pathway in bladder cancer.

## Methods

### Sequence access and retrieval of clinical and somatic data

We obtained approval from the database of Genotypes and Phenotypes (dbGaP) to access exome sequences and germline variant calls from TCGA (accession number phs000178.v9.p8). We downloaded WES files (.bam files) for 104 randomly selected tumor-normal GBM cases from TCGA. For the remaining five TCGA cancers (BLCA, LGG, LUAD, SKCM, STAD), we downloaded Protected Mutation vcf files with somatic and germline variants for entry into the TOBI.vcf pathway indicated in Fig. 1b. We downloaded and analyzed all TCGA Data Matrix cases with Broad Institute-generated Protected Mutation vcf files between July 28, 2015 and September 1, 2015, as well as 226 additional LGG cases downloaded between September 1, 2016 and September 4, 2016. For STAD, 282 cases had available vcf files; 63 cases classified as “hyper-mutated” in TCGA clinical data were excluded from the main analysis. For all six TCGA cancers, clinical data was retrieved from cBioPortal^54^ and publication MAFs from the TCGA Data Matrix provided true somatic variant calls.

We analyzed the WES files (.bam files) for the 92 GBM cases analyzed in Wang et al. 2016. Published somatic calls were used to label true somatic variants.

For pediatric glioma WES sequence files, we obtained approval from the appropriate Data Access Committees (DAC) and downloaded all available sequence files from EGA. Bam files were available for datasets EGAD00001000807^33^ (St. Jude Children’s Research Hospital—Washington University Pediatric Cancer Genome Project Steering Committee) and EGAD00001000706^46^ (ICR DIPG Data Access Committee). Fastq files were available for EGAD00001000792^31^ and EGAD00001000791^22^ (McGill-DKFZ Pediatric Brain Tumour Consortium); samples were mapped to GRCh37.71 using BWA 0.7.12^55^ before variant calling. Clinical data was retrieved from supplementary tables. Published somatic variant calls were used to label true somatic variants for the 74 paired samples; only experimentally validated somatic mutations from Wu et al. 2014^10^ were included.

For 1000 Genomes Project^26^ samples, phase 3 bam files were downloaded from the public FTP site for the first 99 “mapped” samples listed in ftp://ftp.1000genomes.ebi.ac.uk/vol1/ftp/alignment_indices/20130502.exome.alignment.index, as well as sample NA11994, which was previously reported to have a germline variant in *TP53* (R273H).^24^

All GBM, pediatric glioma, and 1000 Genomes Project bam files went through the TOBI.bam pathway indicated in Fig. 1b.

### Variant calling and annotation

Bam files were analyzed with Samtools and Bcftools^56^ to call variants, excluding variants with mapping quality lower than 10.

Variants were annotated using SnpEff^57^ and SnpSift with dbSNP build 144, Cosmic v74, and dbNSFP v2.4 databases.^58^ We also annotated the variants with an in-house database of common mutations in 219 normal WES cases (“Meganormal” database).

### Filtering

Filters thresholds were selected based on preliminary analysis of GBM samples. We applied two main filters on the variants: (1) Technical filter and (2) Biological filter. The technical filter retained all variants with either a quality score from Bcftools^56^ greater than 60 or variant depth higher than 10 on both strands. These filters retained a high fraction of true somatic mutations in known driver genes (e.g. *EGFR*, which had good depth but a QUAL score ≤60) while removing many low quality variants. Variants with sample VAF (the number of sequencing reads supporting a variant nucleotide divided by the total number of sequencing reads at that genomic position) less than 1% were removed. We also removed the variants that had low mapping quality (mq < 40), and had strand bias, map quality bias, and tail distance bias with the *p*-values below 0.01. In the biological filter, we removed common SNPs (population allele frequency greater than 1% in the 1000 Genome Project populations), as well as variants that were present in our Meganormal database. We also removed the SNPs that were in the dbSNP database, but were not in COSMIC. Variants in intragenic, non-coding exon, and splice-site regions were also filtered. We applied these filters to GBM and pediatric glioma variants.

The TCGA variants in the TOBI.vcf pathway did not have reported per strand depth, mapping quality, and technical biases; thus, we used a modified Technical filter to remove variants with total depth < 10 and QUAL score < = 60. Biological filters were the same across all samples.

### Machine learning

We selected the gradient-boosting algorithm for machine learning given its excellent performance on diverse binary classification problems compared to other supervised learning methods.^25^ This algorithm generates a classification model using an ensemble of decision trees that iteratively learn from the previously misclassified training set observations. Gradient boosting returns a probability that a variant is somatic, which TOBI converts into a binary decision using an optimized probability threshold. TOBI does not use the default threshold probability of 0.5 because that would favor the majority class (in our case, non-somatic mutations), resulting in low sensitivity.^59^ Instead, TOBI selects a probability threshold that maximizes classification performance; the threshold’s potential range is 0.05–0.95 in increments of 0.0375.

For each cancer, TOBI generates an optimum classification model by running a systematic grid search through gradient boosting’s three parameters: number of trees (100, 150, 200), interaction depth (3–7 splits), and shrinkage (constant at 0.1). For each possible combination of these three parameters, TOBI performs five repeats of 5-fold cross-validation on the training set in order to avoid over-fitting to the training set. The large number of training set variants compared to features also avoids overfitting. TOBI finally selects the parameter combination that maximizes average performance across the five repeats as the final classification model.

To select the best model despite the class imbalance, we used the *F*-score as the model performance metric:

1 $$F1 = 2\frac{{{\rm{Precision}} \times {\rm{Recall}}}}{{{\rm{Precision}} + {\rm{Recall}}}} = \frac{{2{\rm{TP}}}}{{2{\rm{TP}} + {\rm{FP + FN}}}}$$

where TP, FP, and FN stand for true positive, false positive, and false negative. Maximizing *F*-score results in maximizing TP while minimizing FP and FN. We also assessed performance by calculating sensitivity, specificity, positive predictive value, negative predictive value, prevalence, accuracy, FPR, false discovery rate (FDR), and AUC. For these calculations, true negatives were those variants that passed all TOBI quality filters, were not published as somatic in source publications, and were not predicted as somatic by TOBI.

Here, we describe the software implementation of gradient boosting. For each cancer, cases were randomly assigned to the training or test set using the sample() function without replacement in R. TOBI then calculated cohort-specific annotations separately for the training and test set (see Supplementary Text for features). Somatic status of training set variants was annotated using a user-supplied list of somatic variants, defined by affected case, genomic position, and variant nucleotide. Next, TOBI used the Caret and gbm packages in R^59^ to perform gradient boosting and generate a classification model. To assess feature importance, relative influence of features was automatically calculated during model generation. Relative influence is a measure of how many times a feature is selected for splitting in all trees in the gradient boosting model, weighted and scaled so that the sum of relative influence of all features equals one hundred.

We defined drivers in Fig. 2 using the list of driver genes provided by the Intogen group.^35^

The rate of somatic SNVs per Mb for each case was calculated using the number of published somatic SNVs, after converting di-nucleotide mutations into single nucleotide components and removing indels. This number was divided by the total Mbs covered in Agilent SureSelect Human All Exon 50 Mb regions.bed file.

### Germline variant analysis and clinical data associations

Germline VAFs were available in Protected Mutation vcf files for five TCGA cancers (BLCA, LGG, LUAD, SKCM, STAD). For tumor-normal pediatric glioma cases, germline VAFs were determined using the SAVI variant caller.^29^ For enrichment of gene sets in FP variants, the Poisson cumulative distribution was calculated for each gene set, with *g* total genes and *n* FP variants in those genes from a cancer dataset with *N* variants found in *G* genes, as the probability of a value greater than $(n - 1)$ with $lambda = \frac{{g*N}}{G}$ using the R *ppois* function: ppois(*n*−1, g*N/G, lower.tail = FALSE). Protein domain names and coordinates from PFAM.^60^

Clinical data was retrieved from supplementary tables for Ped.Glioma patients and using the R *cgdsr* package for TCGA. To standardize nomenclature for reported race across studies, we removed samples with missing or mixed classification (“Asian & White”, “Multiple (NOS)”, “Mixed”, “”, “.”, “N/A”, “Other”, “[Not Evaluated]”, “[Unknown]”), and standardized “BLACK OR AFRICAN AMERICAN” to “black”. Patient counts after standardizing nomenclature are in Supplementary Table 1b. We compared the distribution of diagnosis age for cases with or without SLG variants using the Wilcoxon–Mann–Whitney test in R, wilcox.test().

g:Profiler^43^ analysis of BLCA nonsense SLG variants was run using defaults (Significant only; Hierarchical sorting; Numeric IDs treated as: WIKIGENE_ACC; Significance threshold: g:SCS threshold; Statistical domain size: Only annotated genes.) Multiple testing correction for *p*-values calculated using the ontology-focused correction method g:SCS as previously described.^43^ FA pathway in Fig. 5c modified from KEGG FA pathway and Ceccaldi et al. 2016.^61^ CNV data was retrieved from cBioPortal.

### Mutation spectra and signatures

Non-negative matrix factorization approach developed by Alexandrov et al. was applied to infer the mutational signatures of Bladder cancer. The software package was downloaded from http://www.mathworks.com/matlabcentral/fileexchange/38724.

### Comparison to other techniques

In order to compare results from TOBI to other techniques, we ran six GBM samples and six Pediatric Glioma samples through SomVarIUS (Smith et al., 2015) and VNC (Hiltemann et al. 2015) and compared their results to TOBI.

Code for SomVarIUS was obtained through their github page (https://github.com/kylessmith/SomVarIUS). To build the reference database, we supplied an hg19 dbSNP bed to generate the required pickle file. The call_mutations command was then run with the following options:

germ_pos All.filt.pickle --dbsnp_bed All.filt.bed --min_reads 10 --min_support 4 --min_af 0.05 --min_pvalue 0.0001 --min_fr 0.8 --min_qual 25 --min_se 0.999 --min_hetero 0.95 --min_mapq 55 --ref_filter True --dbsnp_bed All.filt.bed --min_baseq 13 --binom False --hapmap All.filt.pickle.

Code VNC was obtained through their github page (https://github.com/shiltemann/Virtual-Normal-Correction). To build the reference virtual normal, 433 CG-sequenced normal exomes were downloaded from 1000Genomes(ftp://ftp.1000genomes.ebi.ac.uk/vol1/ftp/historical_data/former_toplevel/complete_genomics_indices/20130725.cg_data.untar.index). The virtual-normal-correction-smallvariants.sh script was run using the following commands:

--threshold 1 --threshold_highconf 3

### Data availability statement

All genomic datasets used for analysis come from publically accessible repositories after approval for controlled data access:

The database of Genotypes and Phenotypes (dbGaP: https://www.ncbi.nlm.nih.gov/gap): TCGA (accession number phs000178.v9.p8); Clinical information for TCGA from cBioPortal (http://www.cbioportal.org/);

The Sequence Read Archive (https://www.ncbi.nlm.nih.gov/sra): non-TCGA GBM cases analyzed in Wang et al. 2016 (SRP074425);

The European Genome-phenome Archive (https://www.ebi.ac.uk/ega/): St. Jude Children’s Research Hospital—Washington University Pediatric Cancer Genome Project Steering Committee (EGAD0000100080732), ICR DIPG Data Access Committee (EGAD0000100070645), McGill-DKFZ Pediatric Brain Tumour Consortium (EGAD0000100079231, EGAD0000100079122).

The 1000 Genomes Project phase 3 (ftp://ftp.1000genomes.ebi.ac.uk/vol1/ftp/phase3/).

Publication MAFs for TCGA samples from the TCGA Data Matrix (now the Genomic Data Commons https://gdc.cancer.gov/). Publication MAFs from all other publications acquired from supplemental tables of publications.

### Code availability

The TOBI framework is fully available for academic use on Github (https://github.com/RabadanLab/TOBI). This Github page describes all dependencies and versions. We also have a public Amazon Machine Image (AMI) on Amazon Web Services, that contains all the software, dependencies and reference databases used in this article, and it can be shared upon request.

## Electronic supplementary material

Supplementary Note

Supplementary Figures

Supplementary Table 1

Supplementary Table 2

Supplementary Table 3

Supplementary Table 4

Supplementary Table 5

Supplementary Table 6

Supplementary Table 7

Supplementary Table 8

Supplementary Table 9

Supplementary Table 10

Supplementary Table 11

Supplementary Table 12
