# Supplementary material for: Identification of potentially oncogenic alterations from tumor-only samples reveals Fanconi anemia pathway mutations in bladder carcinomas
Source: NPJ Genom Med. 2017 Oct 3;2:29. doi: 10.1038/s41525-017-0032-5 (PMC5677944; doi:10.1038/s41525-017-0032-5)
Supplement: Supplementary file 1 — Supplementary Note [file 41525_2017_32_MOESM1_ESM.docx]

# Supplementary Text

# This Supplementary Text contains Explanation of all features in Figure 1c, Additional Features used in preliminary GBM analysis, Binning of features in Supp. Fig. 1b, and titles and descriptions of Supplementary Files.

**Explanation of all features in Figure 1c**

(1) “Var. per Gene” is the total number of variants per gene normalized by the number of patients in the cohort, calculated separately for training and testing datasets.

(2) “Num. COSMIC Var.” is the total number of samples in COSMIC with this specific nucleotide variant (“CNT” in COSMIC v74 vcf).^1^

(3) “Allele Frequency” is the variant allele frequency (VAF) in the tumor sample.

(4) “CADD Score” is the Combined Annotation–Dependent Depletion Score, a score of variant deleteriousness integrated from multiple genome annotations. For more details, please see Kircher et al^2^.

(5) “Num. COSMIC Gene” is the total number of COSMIC mutations in a gene.

(6) “Protein Length” is the length of the protein in amino acids.

(7) “VAF score” is the probability of a mutation to be a germline mutation with VAF = 50%. It’s calculated using the binomial distribution:

| $VAF Score=Binom({dp}_{var}, dp_{tot}, 0.5)$ | (1) |
| --- | --- |
|  |  |

where ${dp}_{var}$ and ${dp}_{tot}$ are variant depth and total depth, respectively. The justification is that assuming no copy number variation (CNV), the VAF of germline mutations should be either 50% or 100%. This can be seen in Supp. Fig. 1a where a local minimum ratio of somatic to non_somatic mutations occurs around VAF = 50%. This feature helps identify mutations with a high probability of being germline in cases without CNV.

(8) “Mutability” indicates if a gene is prone to mutation in a normal, non-tumor cohort. Per gene calculation involved counting the total number of mutations per gene in a cohort of 219 normal samples, and dividing by the amino acid length.

(9) “Var. per Case” for a particular gene and a particular sample represents the number of variants in that sample divided by the number of patients in the cohort; cohort indicates either training or testing set.

(10) “Variant Impact” is the predicted effect impact from SnpEff, and it can be “High”, “Moderate”, “Low”, and “Modifier”. For more details, please see Cingolani et al^3^.

**Additional Features used in preliminary GBM analysis**

(11) “Recurrent” is the number of recurrent mutations for the specific variant in the cohort.

(12) “ID” is 1 if the mutation is in no database, 2 if it is both in dbSNP and COSMIC, and 3 if it is only in COSMIC. Mutations that are only in dbSNP where filtered out.

(13) “MutationAssessor is a functional impact of amino-acid substitutions in proteins, such as mutations discovered in cancer or missense polymorphisms. The functional impact is assessed based on evolutionary conservation of the affected amino acid in protein homologs”^4^.

(14) “MutationTaster” is a composition of different scores including evolutionary conservation, splice-site changes, and loss of protein features^5^.

(15) “Polyphen” is a score that predicts possible impact of a mutation on the structure and function of a human protein using physical and comparative considerations^6^.

(16) “SIFT” is a score of the effect of a mutation on the protein structure^7^.

**SUPPLEMENTARY FILES**

**Supplementary Figure 1. Effect of removing individual features or all COSMIC associated features from the TOBI model.** Each box indicates a cancer type. Left of the dashed line indicates performance using the standard TOBI model with all features included; to the right, F-scores after the specified feature is removed from the model. Points represent F-score for five runs with randomly selected training and testing sets from specified race; error bars represent mean +/- s.e.m.

**Supplementary Figure 2. TOBI performance when training and testing set are stratified by patients’ self-reported race**. Each box corresponds to one cancer type. Y-axis shows F-score, x-axis shows reported race of training set used to generate model (20 randomly selected patients) above reported race of test set; number of cases in the race-stratified test set shown within plot area. Self-reported race categories required greater than 20 patients for inclusion as a training set, and a minimum of 5 patients for inclusion as a test set. Points represent F-score for five runs with randomly selected training and testing sets from specified race; error bars represent mean +/- s.e.m.

**Supplementary Figure 3.** **TOBI accuracy and F-score when training and testing set are stratified by institution**. Each box corresponds to one cancer type. Y-axis shows F-score, x-axis shows reported race of training set used to generate model (20 randomly selected patients) above institution of test set; number of cases in the test set shown within plot area. An institution required greater than 20 patients for inclusion as a training set, and a minimum of 5 patients for inclusion as a test set. Points represent performance metric for five runs with randomly selected training and testing sets from specified race; error bars represent mean +/- s.e.m. (a) Stratifying GBM cases analyzed by TCGA or within Wang et al., 2016., excluding TCGA cases within Wang analysis, (b) by TCGA cases versus Wang cases collected and analyzed in Seoul, and (c) by TCGA cases identified as “white” versus Seoul cases. (d) Stratifying Ped.Glioma cases analyzed by PCGP or ICR.

**Supplementary Figure 4.** **Inclusion of 61 STAD cases with hypermutation phenotype does not significantly alter TOBI performance.** p-value from Welch’s Two Sample t-test.

**Supplementary Figure 5. Comparison of performance metrics in cancers analyzed by TOBI. (a)** Histogram of performance across all variants in each case. Metric on top of column; each row is a cancer type. Y-axis: case counts, x-axis: 0 to 1 range of metrics. In each box, the upper number represents that performance metric across all samples and variants in that cancer subtype; the ordered pair represents “(mean, median)” of metric for that patient cohort; dashed line=mean, dotted line= median. (b, c) Sensitivity, specificity, and F-score of variants (b) with VAF 0-100% binned by 5% or (c) VAF 0-20% binned by 1%.

**Supplementary Figure 6 F-score for TOBI prediction on genes binned by recurrence of true somatic mutations after filtering.** Recurrence bins defined as high (>20% of tumors), middle (10-20%) genes, and low (<10%)

**Supplementary Figure 7.** **Comparison of performance metrics in 9 FFPE tumor cases and 161 frozen cases from the LUAD TCGA cohort.** Metric listed on top of box; for each metric, top figure represents FFPE samples, bottom frozen samples. Y-axis of case counts, x-axis represents 0 to 1 range of metrics. In each box, ordered pair represents “(mean, median)” of metric for that patient cohort; dashed line=mean, dotted line= median.

**Supplementary Figure 8. TOBI somatic variant prediction outperforms other methods.** ROC curves comparing somatic variant prediction (synonymous and nonsynonymous) based on TOBI, CADD score, Mutation Assessor, SIFT and MutationTaster.

**Supplementary Figure 9. False positive rate (FPR) in seven cancers compared to false positive rate from 1000Genomes samples**. Distribution of FPR per case for each cancer was compared to the FPR from 100 cases from the 1000Genomes project with no cancer diagnosis. For the seven cancers and patients analyzed in figure 1b,c, FPR was calculated as the number of false positive TOBI somatic calls divided by the total number of true non-somatic variants in each case after filtering; for 1000Genomes samples, false positives were defined as any variant predicted as somatic by TOBI, and FPR calculated by dividing the number of false positives by the total number of variants after filtering. p-value calculated with the two-sided Wilcoxon–Mann–Whitney test.

**Supplementary Figure 10. Age distribution for cases with or without SLG in specified gene sets.** All gene sets except Fanconi anemia genes retrieved from36; Fanconi anemia genes from KEGG map 03460 and hsa03460. Colors consistent with legend in Figure 1b-c. p-value calculated with the two-sided Wilcoxon–Mann–Whitney test; * indicates p<0.01. (a) autosomal dominant cancer-predisposition syndromes (AD genes), (b) 565 cancer genes, (c) Fanconi anemia (FA) pathway.

**Supplementary Figure 11. g:Profiler results on 49 genes with predicted SLG nonsense variants in BLCA.** Analysis run using g:Profiler defaults (Significant only; Hierarchical sorting; Numeric IDs treated as: WIKIGENE_ACC; Significance threshold: g:SCS threshold; Statistical domain size: Only annotated genes.) KEGG results highlighted.

**Supplementary Figure 12. Four somatic signatures for BLCA.** (a) Selection of k=four somatic signatures for BLCA maximizes stability and minimizes error. (b) Somatic signatures from TCGA BLCA cohort (TCGA, Nature 2014), generated using techniques from Alexandrov et al, Nature 2013. Signature 4 resembles the BRCA somatic signature described in Alexandrov**.**

**Supplementary Figure 13. Sensitivity correlates with somatic SNV rate**. (a) Somatic SNV per megabase (Mb) for each cancer type. Vertical axis shows the number of somatic SNV per megabase on a log10 scale. Each point represents a tumor sample, red horizontal lines indicate median value for cancer; cancers ordered by increasing median number of somatic mutations. (b) Same as Figure 2a but cancers are ordered by increasing median number of somatic mutations. (c) Scatterplot of median somatic SNV per Mb versus true positive rate of nonsynonymous variants. Each point is a cancer type. Left panel uses true positive rate from all genes, right panel for driver genes only. P-value for Spearman correlation.

**Supplementary Table 1: Patient cohorts and average F-score based on training set size.** (a) Per cancer, total cases, number paired frozen, paired FFPE, hypermutator, or unpaired cases. Number of cases used in figure 1 and figure 2 onward also indicated. (b) For each cancer type with a training set size from 1 to 50, average F-score from five rounds of TOBI.

**Supplementary Table 2: Effect of reported race on performance.** (a). Patient counts using raw reported race categories. (b) Patient counts after standardizing nomenclature. (c) Performance metrics when training and testing set are stratified by patients’ self-reported race.

**Supplementary Table 3: Performance metrics when training and testing set are stratified by analyzing institution**

**Supplementary Table 4: Performance metrics with additional 61 STAD cases with hypermutation phenotype**

**Supplementary Table 5: Performance metrics for all variants across cancer types.** (a) Separated by cancer subtype, (b) separated by case.

**Supplementary Table 6: TOBI predictions on published somatic variants.**

**Supplementary Table 7: Comparison of performance metrics in 9 FFPE tumor cases and 161 frozen cases from the LUAD TCGA cohort.** (a) Performance in FFPE vs. frozen cohort. (b) Performance by FFPE case.

**Supplementary Table 8: Comparison of performance by TOBI, SomVarIUS, and Virtual Normal Correction on six GBM and six Ped.Glioma samples**.

**Supplementary Table 9: Gene set lists for autosomal dominant cancer-predisposition (AD genes), 565 cancer genes, and Fanconi anemia (FA) pathway**

**Supplementary Table 10: Counts of SLG variants across cancers.**

**Supplementary Table 11: Counts of Fanconi anemia alterations in BLCA.** Pathway modified from KEGG FA pathway and Ceccaldi et al 2016^8^.

**Supplementary Table 12: Comparison of variant quality cutoffs used in TOBI and MuTect.**

**References**

1. Forbes, S. A. *et al.* COSMIC: exploring the world’s knowledge of somatic mutations in human cancer. *Nucleic Acids Res.* **43,** D805–D811 (2015).

2. Kircher, M. *et al.* A general framework for estimating the relative pathogenicity of human genetic variants. *Nat. Genet.* **46,** 310–315 (2014).

3. Cingolani, P. *et al.* A program for annotating and predicting the effects of single nucleotide polymorphisms, SnpEff: SNPs in the genome of Drosophila melanogaster strain w1118; iso-2; iso-3. *Fly (Austin)* **6,** 80–92

4. Reva, B., Antipin, Y. & Sander, C. Predicting the functional impact of protein mutations: application to cancer genomics. *Nucleic Acids Res.* **39,** e118 (2011).

5. Schwarz, J. M., Rödelsperger, C., Schuelke, M. & Seelow, D. MutationTaster evaluates disease-causing potential of sequence alterations. *Nat. Methods* **7,** 575–6 (2010).

6. Adzhubei, I. A. *et al.* A method and server for predicting damaging missense mutations. *Nat. Methods* **7,** 248–9 (2010).

7. Kumar, P., Henikoff, S. & Ng, P. C. Predicting the effects of coding non-synonymous variants on protein function using the SIFT algorithm. *Nat. Protoc.* **4,** 1073–81 (2009).

8. Ceccaldi, R., Sarangi, P. & D’Andrea, A. D. The Fanconi anaemia pathway: new players and new functions. *Nat. Rev. Mol. Cell Biol.* **17,** 337–349 (2016).
