# Supplementary material for: Identification of potentially oncogenic alterations from tumor-only samples reveals Fanconi anemia pathway mutations in bladder carcinomas
Source: NPJ Genom Med. 2017 Oct 3;2:29. doi: 10.1038/s41525-017-0032-5 (PMC5677944; doi:10.1038/s41525-017-0032-5)
Supplement: Supplementary file 2 — Supplementary Figures [file 41525_2017_32_MOESM2_ESM.pdf]

Supp. Fig. 1. Effect of removing individual features or all COSMIC associated features from the TOBI model. Each box indicates a cancer type. Left of the dashed line indicates performance using the standard TOBI model with all features included; to the right, F-scores after the specified feature is removed from the model. Points represent F-score for five runs with randomly selected training and testing sets from specified race; error bars represent mean +/- s.e.m.

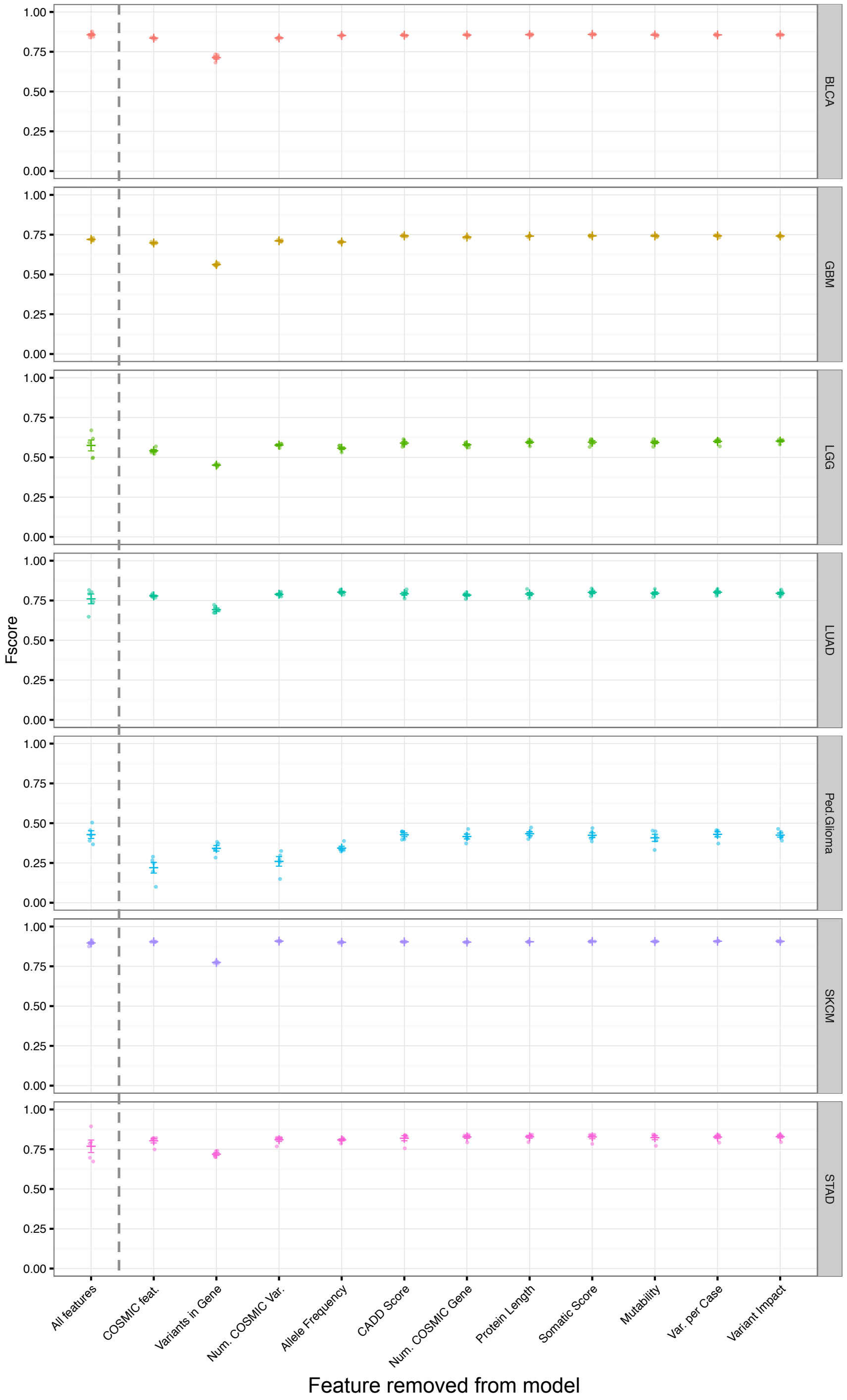

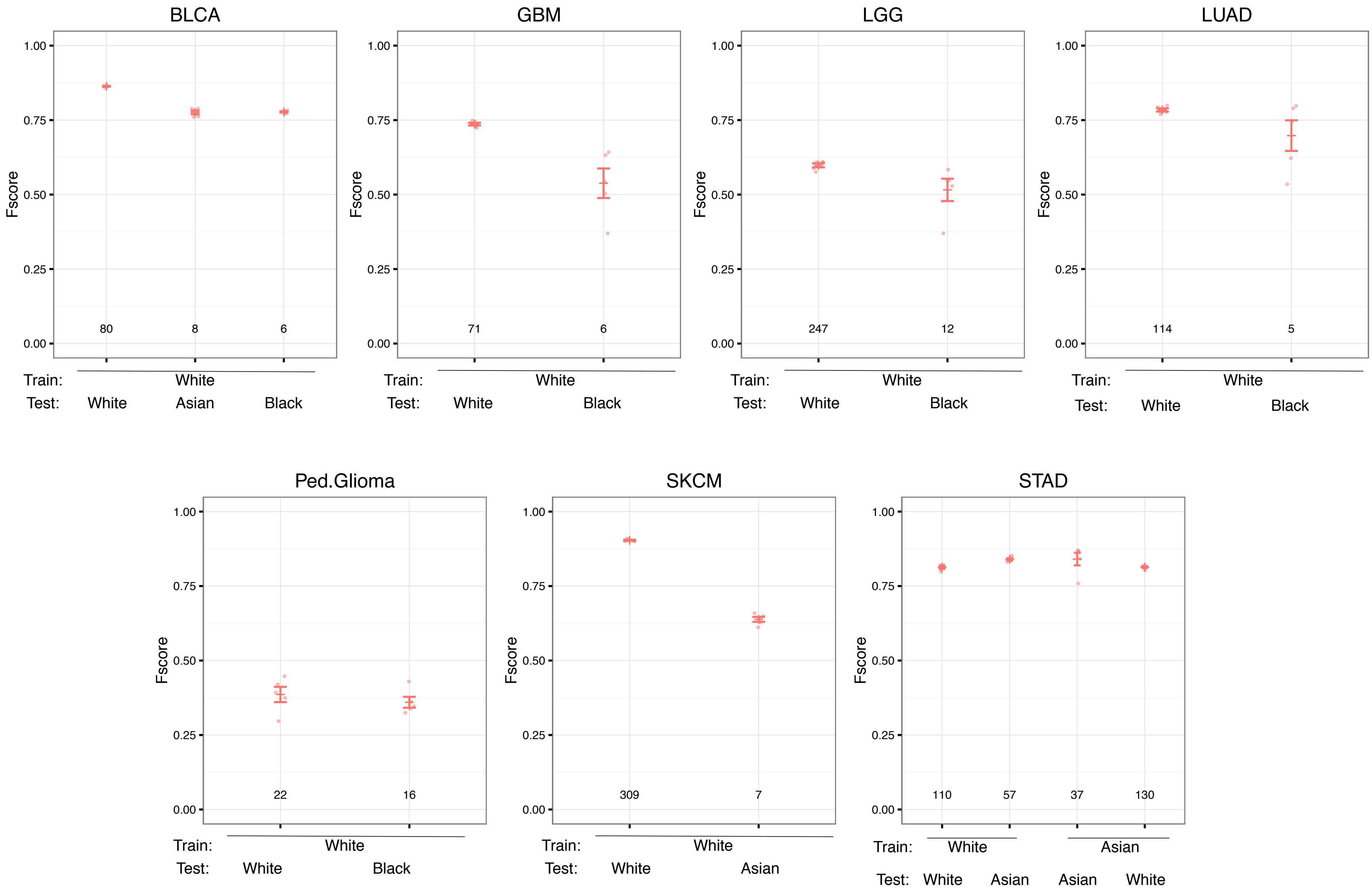

Supp. Fig. 2. TOBI performance when training and testing set are stratified by patients' self-reported race. Each box corresponds to one cancer type. Y-axis shows F-score, x-axis shows reported race of training set used to generate model (20 randomly selected patients) above reported race of test set; number of cases in the race-stratified test set shown within plot area. Self-reported race categories required greater than 20 patients for inclusion as a training set, and a minimum of 5 patients for inclusion as a test set. Points represent F-score for five runs with randomly selected training and testing sets from specified race; error bars represent mean +/- s.e.m.

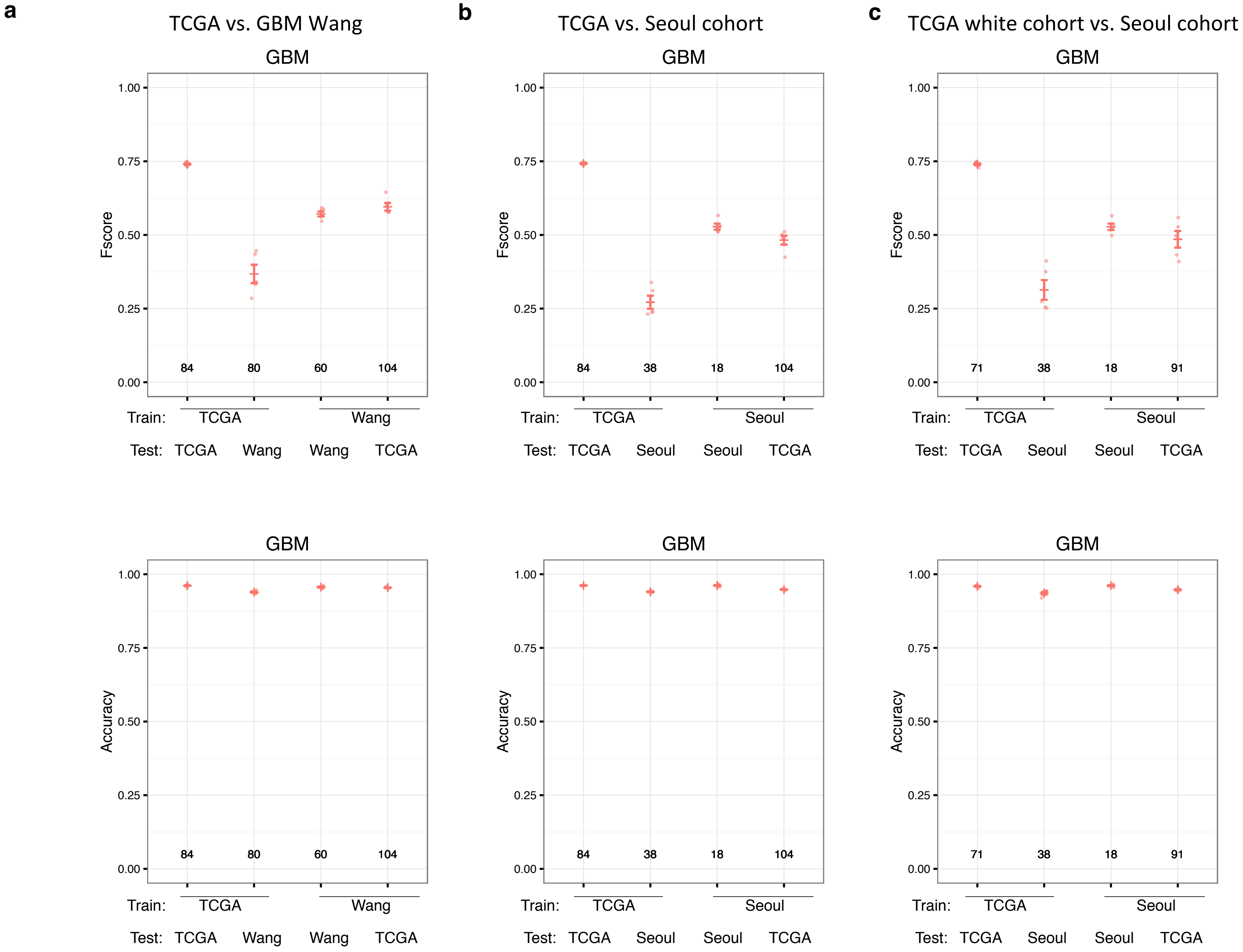

Supp. Fig. 3. TOBI accuracy and Fscore when training and testing set are stratified by institution.

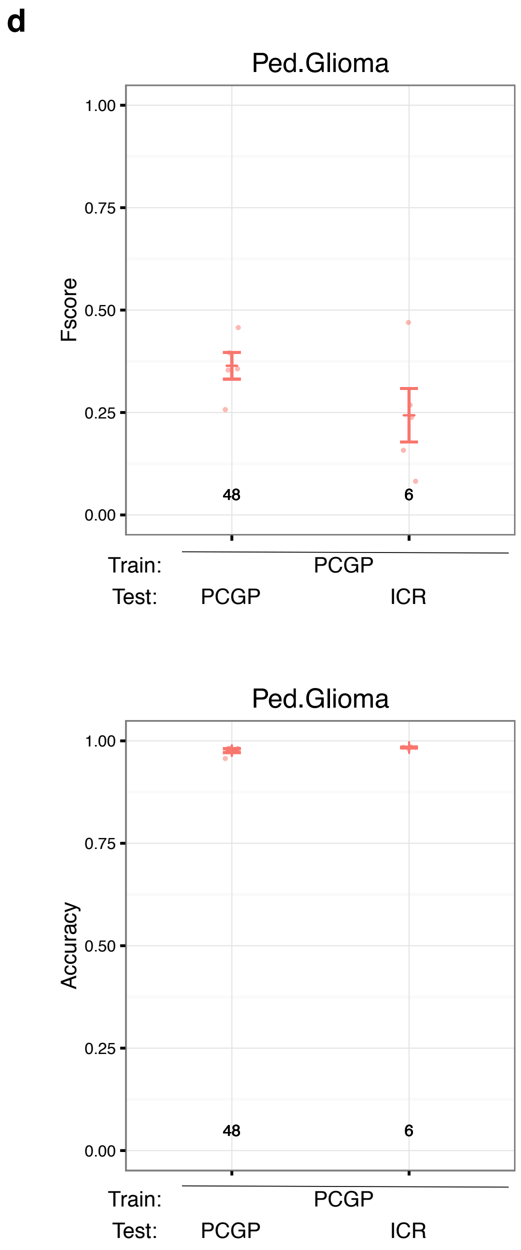

Supp. Fig. 3. TOBI accuracy and Fscore when training and testing set are stratified by institution. Each box corresponds to one cancer type. Y-axis shows F-score, x-axis shows reported race of training set used to generate model (20 randomly selected patients) above institution of test set; number of cases in the test set shown within plot area. An institution required greater than 20 patients for inclusion as a training set, and a minimum of 5 patients for inclusion as a test set. Points represent performance metric for five runs with randomly selected training and testing sets from specified race; error bars represent mean +/- s.e.m. (a) Stratifying GBM cases analyzed by TCGA or within Wang et al., 2016., excluding TCGA cases within Wang analysis, (b) by TCGA cases versus Wang cases collected and analyzed in Seoul, and (c) by TCGA cases identified as “white” versus Seoul cases. (d) Stratifying Ped.Glioma cases analyzed by PCGP or ICR.

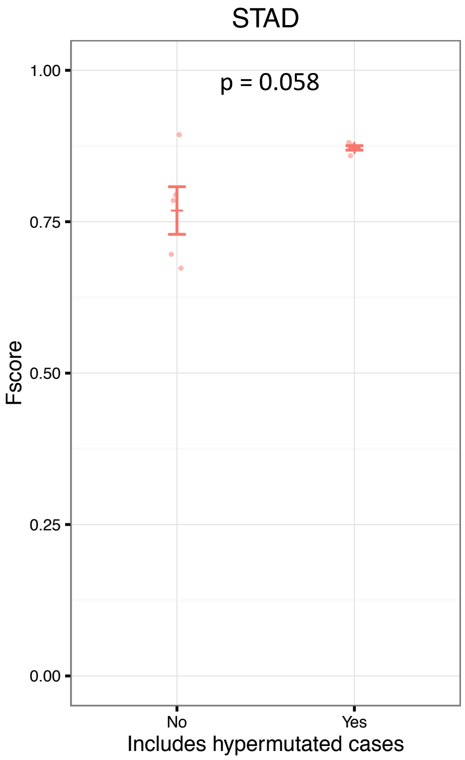

Supp. Fig. 4. Inclusion of 61 STAD cases with hypermutation phenotype does not significantly alter TOBI performance. p-value from Welch's Two Sample t-test.

Supp. Fig. 5. Comparison of performance metrics in cancers analyzed by TOBI. (a) Histogram of performance across all variants in each case. Metric on top of column; each row is a cancer type. Y-axis: case counts, x-axis: 0 to 1 range of metrics. In each box, the upper number represents that performance metric across all samples and variants in that cancer subtype; the ordered pair represents "(mean, median)" of metric for that patient cohort; dashed line=mean, dotted line= median. **(b, c)** Sensitivity, specificity, and F-score of variants (b) with VAF 0-100% binned by 5% or (c) VAF 0-20% binned by 1%.

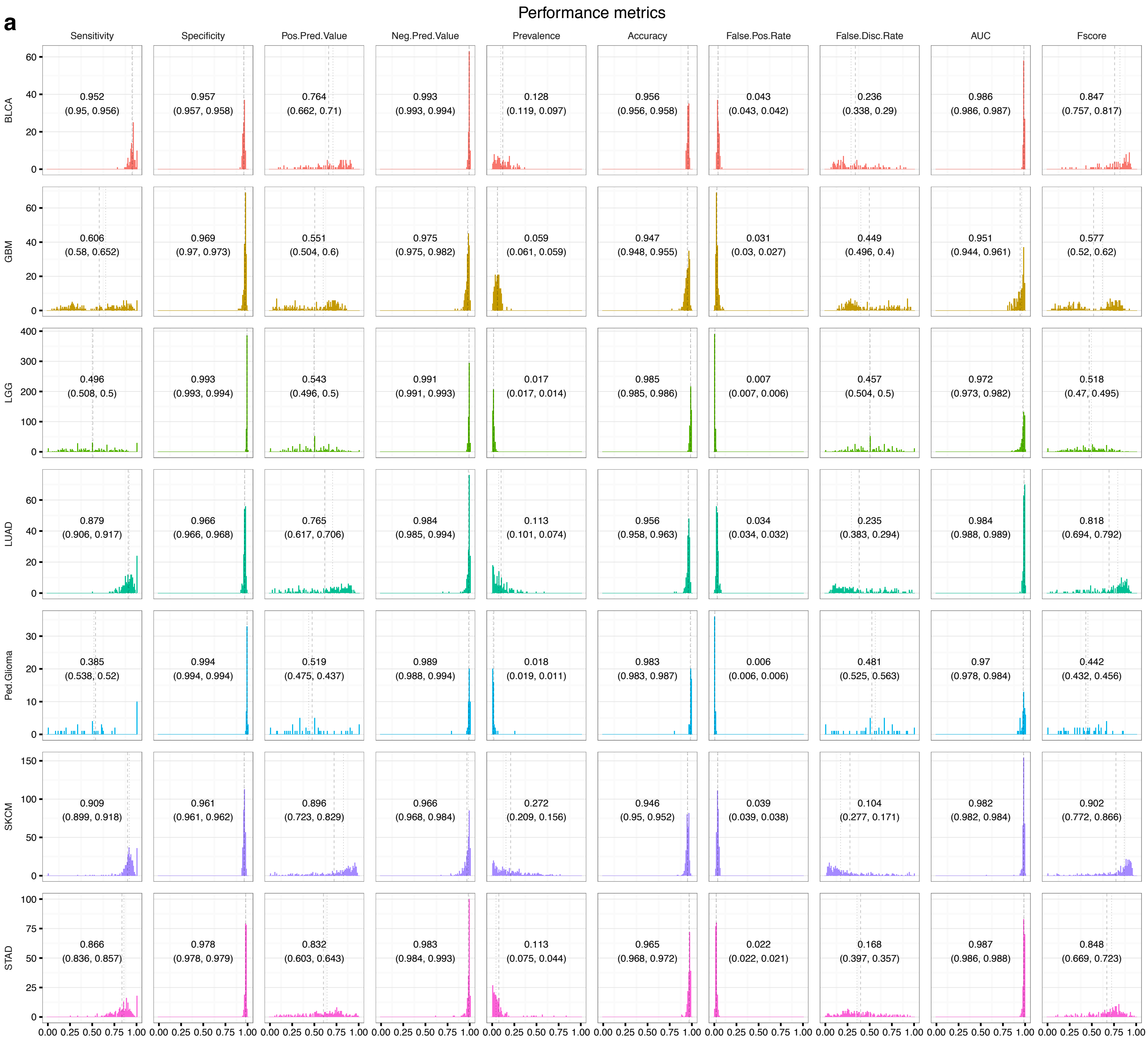

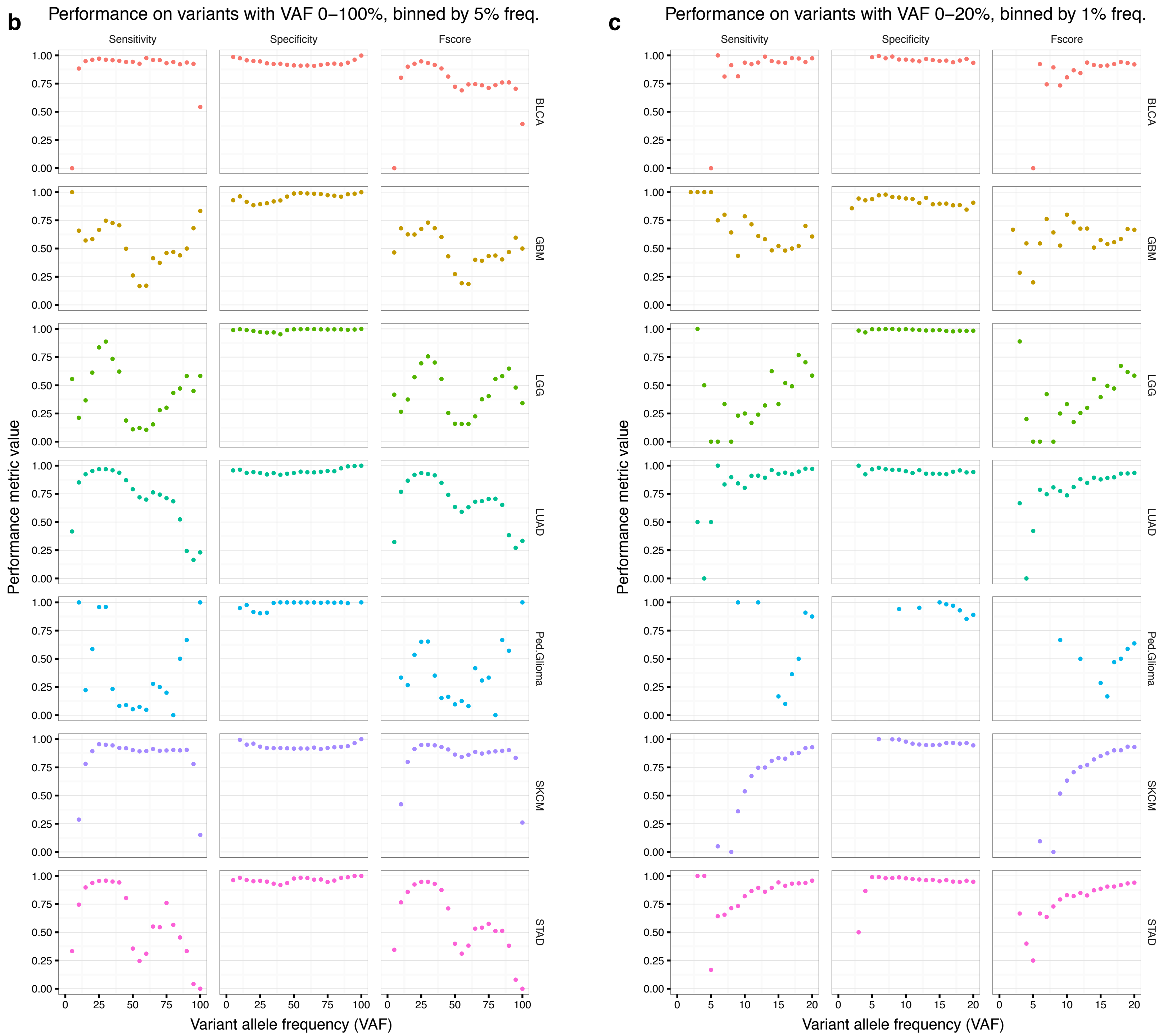
b
Performance on variants with VAF 0–100%, binned by 5% freq.
c
Performance on variants with VAF 0–20%, binned by 1% freq.
Sensitivity
Specificity
Fscore
BLCA
GBM
LGG
LUAD
Ped.Glioma
SKCM
STAD
Performance metric value
Variant allele frequency (VAF)

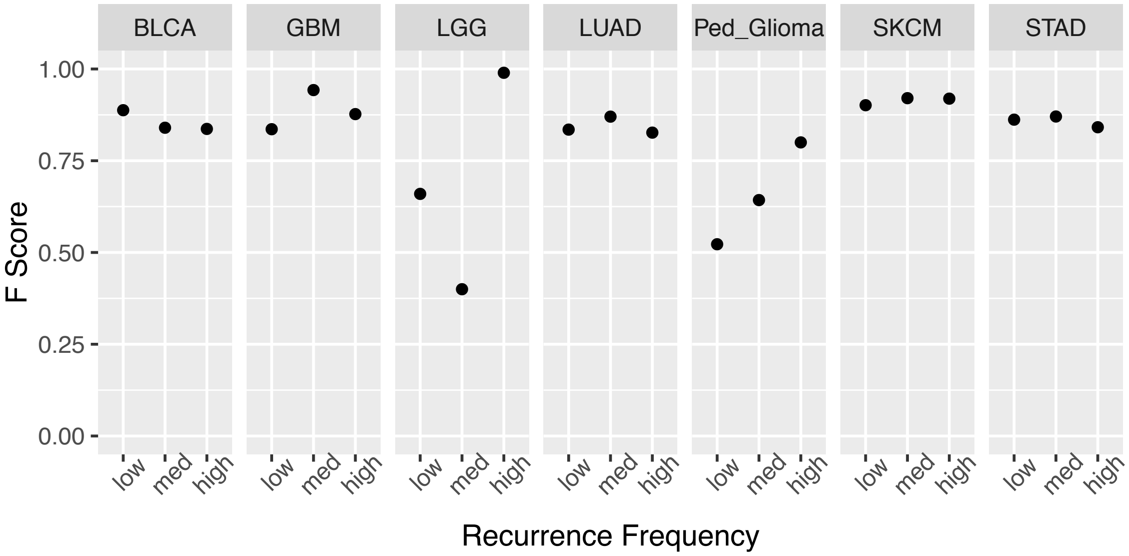

Supp. Fig. 6. F-score for TOBI prediction on genes binned by recurrence of true somatic mutations after filtering. Recurrence bins defined as high (>20% of tumors), middle (10-20%) genes, and low (<10%)

Supp. Fig. 7. Comparison of performance metrics in 9 FFPE tumor cases and 161 frozen cases from the LUAD TCGA cohort. Metric listed on top of box; for each metric, top figure represents FFPE samples, bottom frozen samples. Y-axis of case counts, x-axis represents 0 to 1 range of metrics. In each metrics. In each box, ordered pair represents “(mean, median)” of metric for that patient cohort; dashed line=mean, dotted line= median.

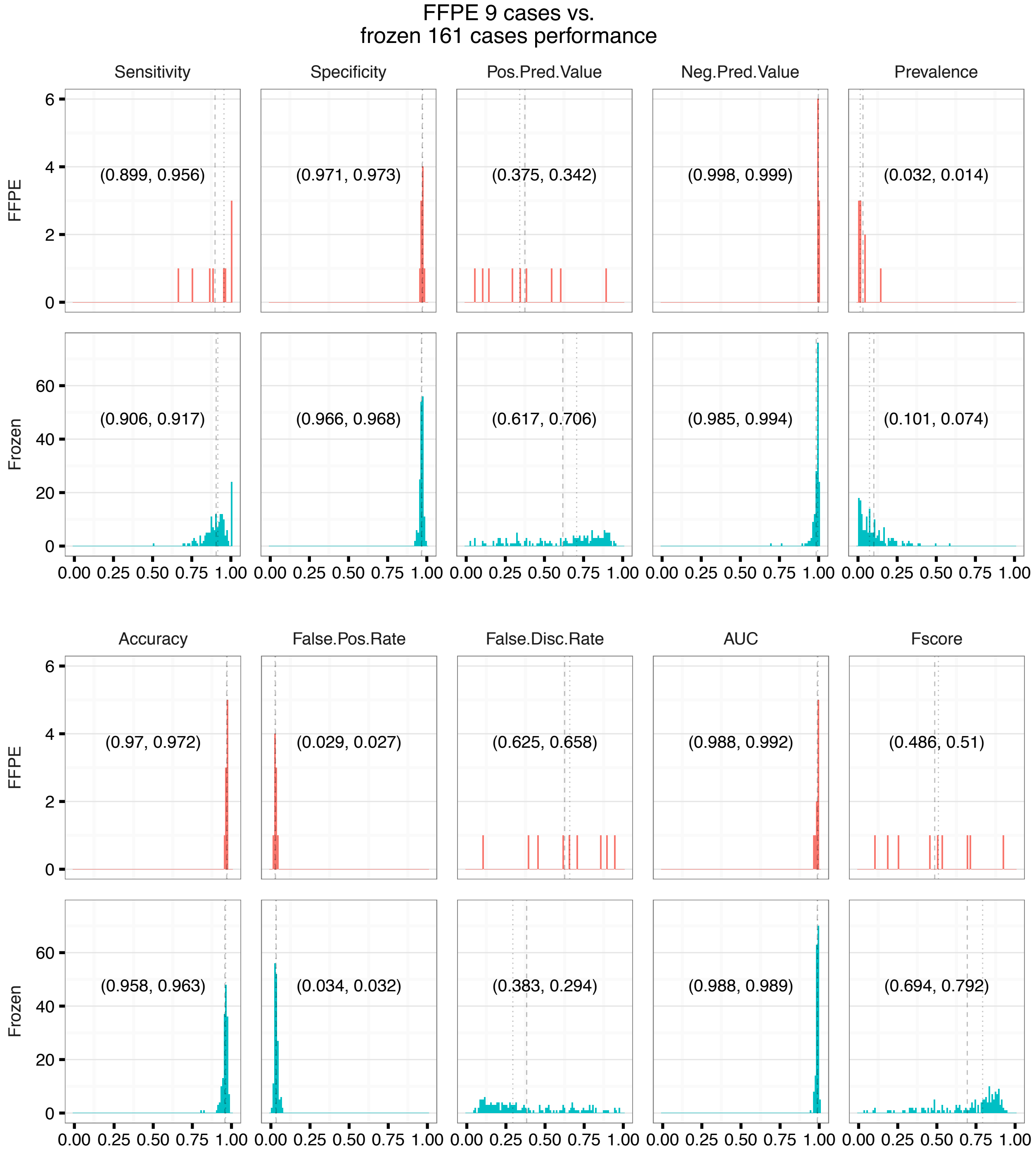

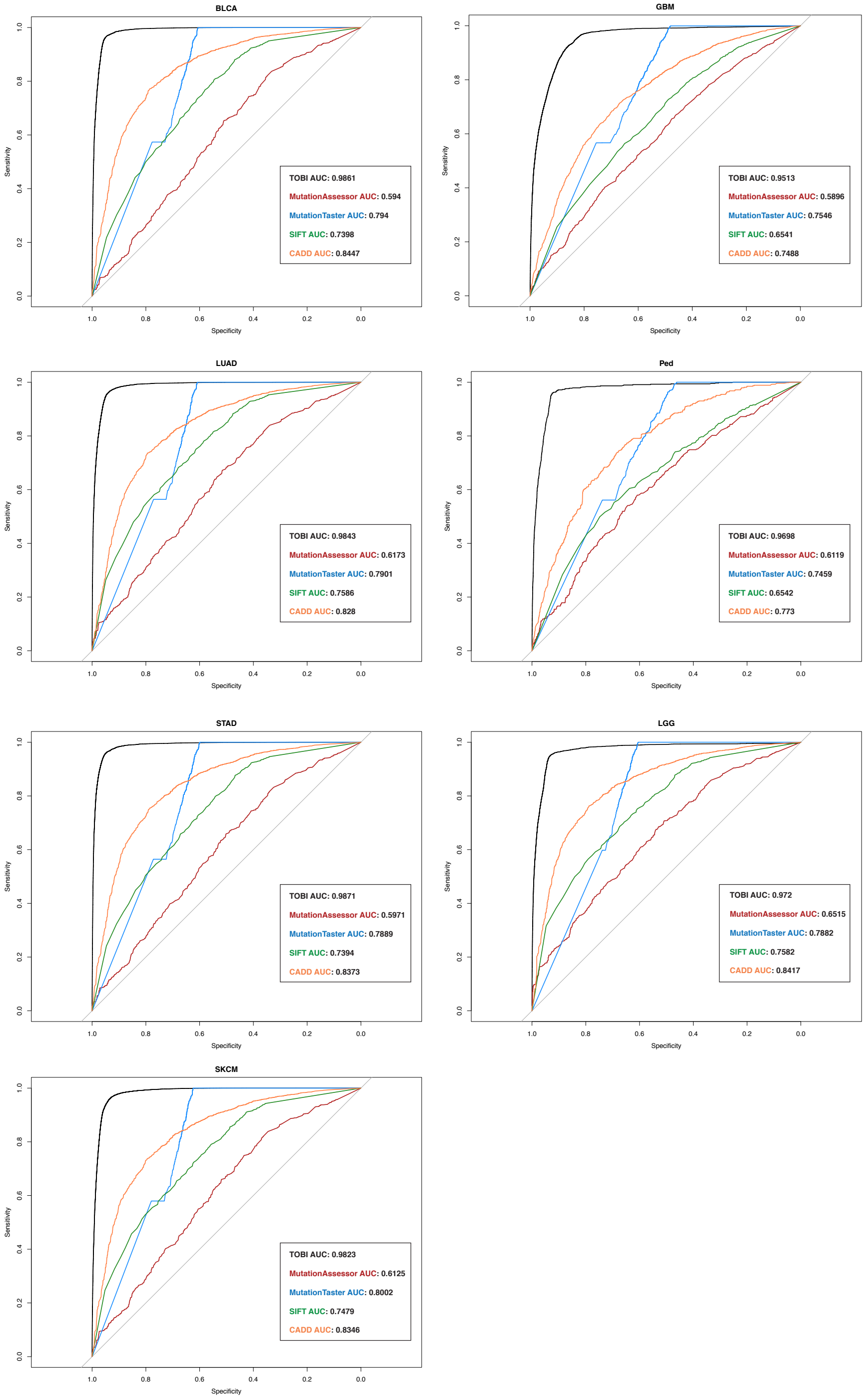

Supp. Fig 8. TOBI somatic variant prediction outperforms other methods. ROC curves comparing somatic variant prediction (synonymous and nonsynonymous) based on TOBI, CADD score, Mutation Assessor, SIFT and MutationTaster.

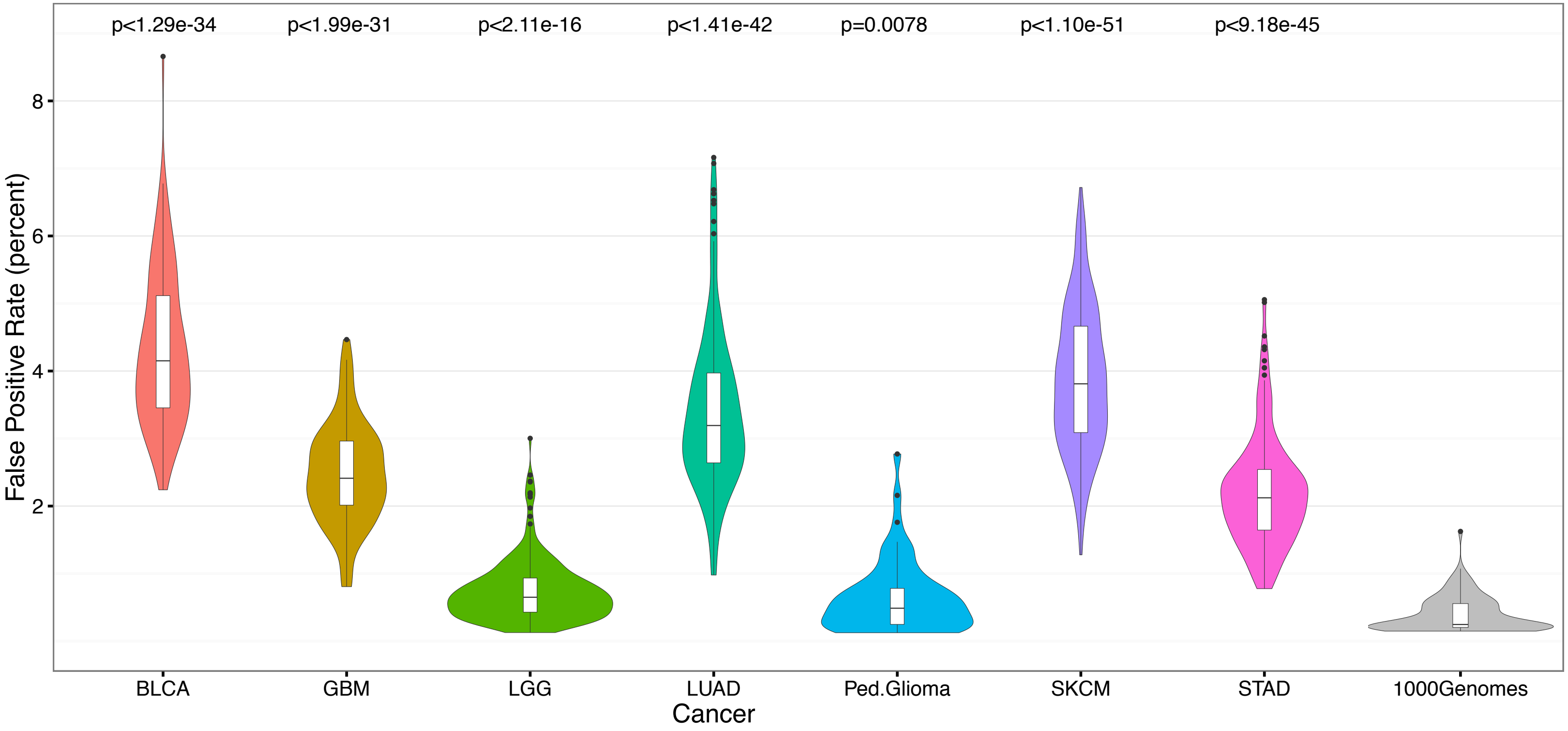

Supplementary Figure 9. False positive rate (FPR) in seven cancers compared to false positive rate from 1000Genomes samples. Distribution of FPR per case for each cancer was compared to the FPR from 100 cases from the 1000Genomes project with no cancer diagnosis. For the seven cancers and patients analyzed in figure 1b,c, FPR was calculated as the number of false positive TOBI somatic calls divided by the total number of true non-somatic variants in each case after filtering; for 1000Genomes samples, false positives were defined as any variant predicted as somatic by TOBI, and FPR calculated by dividing the number of false positives by the total number of variants after filtering. LGG includes original 266 test cases. p-value calculated with the two-sided Wilcoxon–Mann–Whitney test.

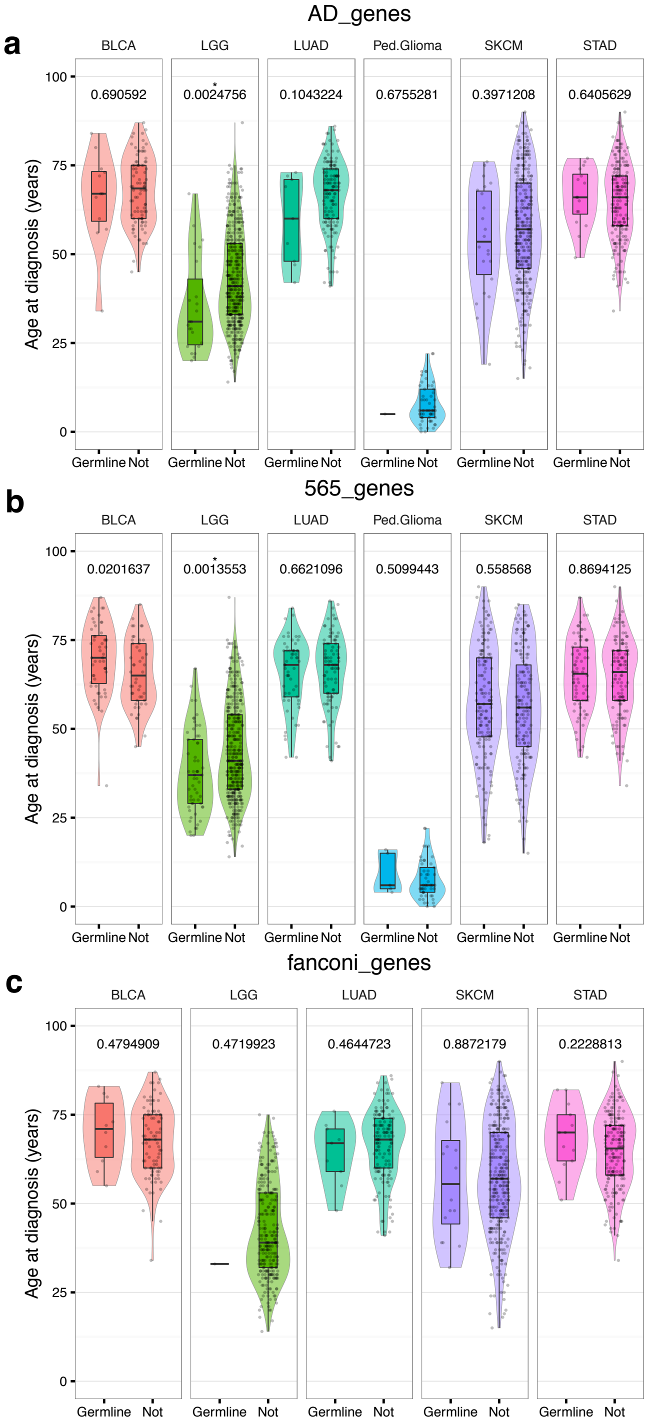

Supplementary Figure 10. **Age distribution for cases with or without SLG in specified gene sets.** All gene sets except Fanconi anemia genes retrieved from36; Fanconi anemia genes from KEGG map 03460 and hsa03460. Colors consistent with legend in Fig. 1b-c. p-value calculated with the two-sided Wilcoxon–Mann–Whitney test; * indicates p<0.01. **(a)** autosomal dominant cancer-predisposition syndromes (AD genes), **(b)** 565 cancer genes, **(c)** Fanconi anemia (FA) pathway.

Human Phenotype Ontology

source	term name	term ID	n. of term genes	n. of query genes	n. of common genes	corrected p-value	BRCA2	CARD10	CCDC86	CENPO	CNGA1	DNAH7	DNMBP	FAM83H	FANCD2	FANCM	GLYATL1	H2BFM	HELQ	KIF15	MC1R	MYO5B	NUDT19	NUFIP1	OLFM3	OLFML2B	OR10G8	OR1K1	OR2V3	PFAS	PGLS	PNMT	PPEF2	PTH2R	SAMD9L	SCN1A	SHPK	SLFN13	SPSB2	SRBD1	SUCNR1	SYNJ2	TCP11	TDO2	TP53I3	TRIM45	TUBB3	ZNF169	ZNF433	ZNF44	ZNF799
hp	Lung segmentation defects	HP:0006525	19	9	3	9.38e-03	h								h	h																																			
hp	Aplasia of the fingers	HP:0009380	81	9	4	2.56e-02	h								h	h																															h				
hp	Hypopigmented skin patches	HP:0001053	92	9	4	4.24e-02	h								h	h					h																														
hp	Vitiligo	HP:0001045	24	9	3	1.95e-02	h								h	h																																			
hp	Clubbing of toes	HP:0100760	26	9	3	2.49e-02	h								h	h																																			
hp	Aplasia/Hypoplasia of the uvula	HP:0010293	18	9	3	7.91e-03	h								h	h																																			
hp	Chromsome breakage	HP:0040012	18	9	3	7.91e-03	h								h	h																																			
hp	Chromosomal breakage induced by crosslinking agents	HP:0003221	17	9	3	6.60e-03	h								h	h																																			
hp	Anemic pallor	HP:0001017	17	9	3	6.60e-03	h								h	h																																			
hp	Biliary atresia	HP:0005912	21	9	3	1.28e-02	h								h	h																																			
hp	Abnormality of the cell cycle	HP:0011018	17	9	3	6.60e-03	h								h	h																																			
hp	Prolonged G2 phase of cell cycle	HP:0003214	17	9	3	6.60e-03	h								h	h																																			
hp	Ureteral duplication	HP:0000073	31	9	3	4.28e-02	h								h	h																																			
hp	Abnormality of the renal collecting system	HP:0004742	26	9	3	2.49e-02	h								h	h																																			
hp	Duplicated collecting system	HP:0000081	23	9	3	1.71e-02	h								h	h																																			
hp	Abnormality of nucleobase metabolism	HP:0010932	27	9	3	2.80e-02	h								h	h																																			
hp	Abnormality of pyrimidine metabolism	HP:0004353	24	9	3	1.95e-02	h								h	h																																			
hp	Abnormality of DNA repair	HP:0003254	24	9	3	1.95e-02	h								h	h																																			
hp	Deficient excision of UV-induced pyrimidine dimers in DNA	HP:0003213	17	9	3	6.60e-03	h								h	h																																			
hp	Esophageal atresia	HP:0002032	28	9	3	3.13e-02	h								h	h																																			
hp	Absent radius	HP:0003974	32	9	3	4.72e-02	h								h	h																																			
hp	Complete duplication of phalanx of hand	HP:0009998	21	9	3	1.28e-02	h								h	h																																			
hp	Complete duplication of thumb phalanx	HP:0009943	21	9	3	1.28e-02	h								h	h																																			
hp	Abnormality of the carotid arteries	HP:0005344	31	9	3	4.28e-02	h								h	h																																			
hp	Multiple cafe-au-lait spots	HP:0007565	21	9	3	1.28e-02	h								h	h																																			
hp	Medulloblastoma	HP:0002885	30	9	3	3.87e-02	h								h	h																																			
hp	Abnormality of the ileum	HP:0001549	25	9	3	2.21e-02	h								h	h																																			
hp	Meckel diverticulum	HP:0002245	23	9	3	1.71e-02	h								h	h																																			

Biological pathways (KEGG)

source	term name	term ID	n. of term genes	n. of query genes	n. of common genes	corrected p-value	BRCA2	CARD10	CCDC86	CENPO	CNGA1	DNAH7	DNMBP	FAM83H	FANCD2	FANCM	GLYATL1	H2BFM	HELQ	KIF15	MC1R	MYO5B	NUDT19	NUFIP1	OLFM3	OLFML2B	OR10G8	OR1K1	OR2V3	PFAS	PGLS	PNMT	PPEF2	PTH2R	SAMD9L	SCN1A	SHPK	SLFN13	SPSB2	SRBD1	SUCNR1	SYNJ2	TCP11	TDO2	TP53I3	TRIM45	TUBB3	ZNF169	ZNF433	ZNF44	ZNF799
keg	Fanconi anemia pathway	KEGG:03460	54	21	3	2.90e-02	k								k	k																																			

Online Mendelian Inheritance in Man

source	term name	term ID	n. of term genes	n. of query genes	n. of common genes	corrected p-value	BRCA2	CARD10	CCDC86	CENPO	CNGA1	DNAH7	DNMBP	FAM83H	FANCD2	FANCM	GLYATL1	H2BFM	HELQ	KIF15	MC1R	MYO5B	NUDT19	NUFIP1	OLFM3	OLFML2B	OR10G8	OR1K1	OR2V3	PFAS	PGLS	PNMT	PPEF2	PTH2R	SAMD9L	SCN1A	SHPK	SLFN13	SPSB2	SRBD1	SUCNR1	SYNJ2	TCP11	TDO2	TP53I3	TRIM45	TUBB3	ZNF169	ZNF433	ZNF44	ZNF799
omi	RETINITIS PIGMENTOSA 49; RP49	OMIM:613756	1	8	1	4.99e-02					om																																								
omi	ALBINISM, OCULOCUTANEOUS, TYPE II; OCA2;;OCULOCUTANEOUS ALBINISM, TYPE II;;OCUL ...	OMIM:203200	1	8	1	4.99e-02															om																														
omi	Fanconi Anemia	OMIM:227650	16	8	2	1.22e-02	om								om																																				
omi	FANCONI ANEMIA, COMPLEMENTATION GROUP D2; FANCD2;;FAD2;;FANCONI ANEMIA, COMP ...	OMIM:227646	1	8	1	4.99e-02									om																																				
omi	FANCONI ANEMIA, COMPLEMENTATION GROUP D1; FANCD1;;FAD1	OMIM:605724	1	8	1	4.99e-02	om																																												
omi	INCREASED ANALGESIA FROM KAPPA-OPIOID RECEPTOR AGONIST, FEMALE-SPECIFIC	OMIM:613098	1	8	1	4.99e-02															om																														
omi	AMELOGENESIS IMPERFECTA, TYPE III; AI3;;AMELOGENESIS IMPERFECTA, HYPOCALCIFICAT ...	OMIM:130900	1	8	1	4.99e-02								om																																					
omi	DIARRHEA 2, WITH MICROVILLUS ATROPHY; DIAR2;;MICROVILLUS INCLUSION DISEASE; MVI ...	OMIM:251850	1	8	1	4.99e-02																om																													
omi	SKIN/HAIR/EYE PIGMENTATION, VARIATION IN, 2; SHEP2;;RED HAIR COLOR; RHC;;BLOND ...	OMIM:266300	1	8	1	4.99e-02															om																														
omi	PANCREATIC CANCER, SUSCEPTIBILITY TO, 2;;PNCA2	OMIM:613347	1	8	1	4.99e-02	om																																												
omi	GENERALIZED EPILEPSY WITH FEBRILE SEIZURES PLUS, TYPE 2; GEFSP2;;GEFS+, TYPE 2; ...	OMIM:604403	1	8	1	4.99e-02																														om															
omi	BREAST-OVARIAN CANCER, FAMILIAL, SUSCEPTIBILITY TO, 2; BROVCA2BREAST CANCER, FA ...	OMIM:612555	1	8	1	4.99e-02	om																																												
omi	GLIOMA SUSCEPTIBILITY 3; GLM3	OMIM:613029	1	8	1	4.99e-02	om																																												
omi	MIGRAINE, FAMILIAL HEMIPLEGIC, 3; FHM3	OMIM:609634	1	8	1	4.99e-02																														om															
omi	MELANOMA, CUTANEOUS MALIGNANT, SUSCEPTIBILITY TO, 5; CMM5	OMIM:613099	1	8	1	4.99e-02															om																														

Regulatory motifs in DNA (TRANSFAC TFBS)

source	term name	term ID	n. of term genes	n. of query genes	n. of common genes	corrected p-value	BRCA2	CARD10	CCDC86	CENPO	CNGA1	DNAH7	DNMBP	FAM83H	FANCD2	FANCM	GLYATL1	H2BFM	HELQ	KIF15	MC1R	MYO5B	NUDT19	NUFIP1	OLFM3	OLFML2B	OR10G8	OR1K1	OR2V3	PFAS	PGLS	PNMT	PPEF2	PTH2R	SAMD9L	SCN1A	SHPK	SLFN13	SPSB2	SRBD1	SUCNR1	SYNJ2	TCP11	TDO2	TP53I3	TRIM45	TUBB3	ZNF169	ZNF433	ZNF44	ZNF799
tf	Factor: ZNF224; motif: NAATGCYAATCY; match class: 0	TF:M06871_0	334	45	6	4.08e-02																tf	tf	tf										tf														tf		tf	

Supplementary Figure 11. g:Profiler results on 49 genes with predicted SLG nonsense variants in BLCA. Analysis run using g:Profiler defaults (Significant only; Hierarchical sorting; Numeric IDs treated as: WIKIGENE_ACC; Significance threshold: g:SCS threshold; Statistical domain size: Only annotated genes.) KEGG results highlighted.

a

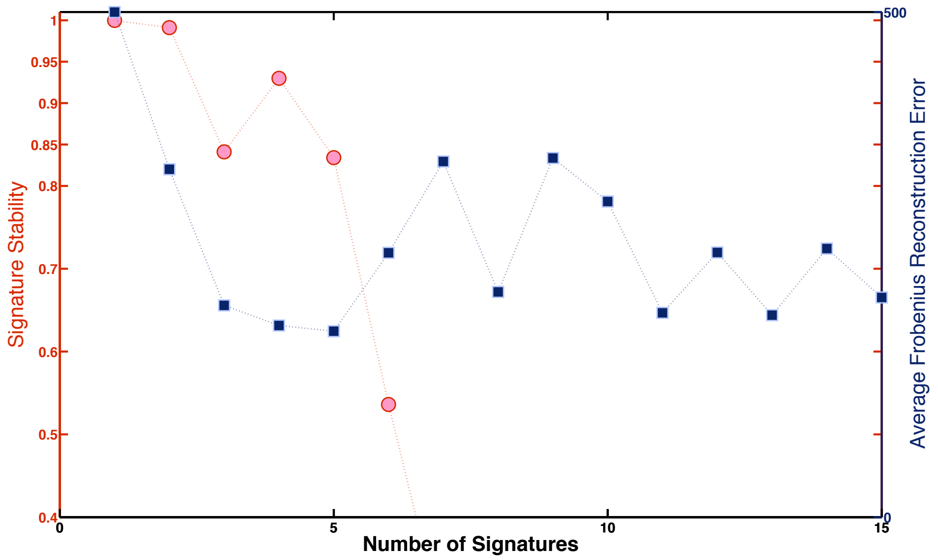

Supp. Fig. 12. Four somatic signatures for BLCA. (a) Selection of k=four somatic signatures for 130 BLCA cases maximizes stability and minimizes error. (b) Somatic signatures from TCGA BLCA cohort (TCGA, Nature 2014), generated using techniques from Alexandrov et al, Nature 2013. Signature 4 resembles the BRCA1/2 somatic signature described in Alexandrov.

b

Signatures in 130 BLCA cases from TCGA

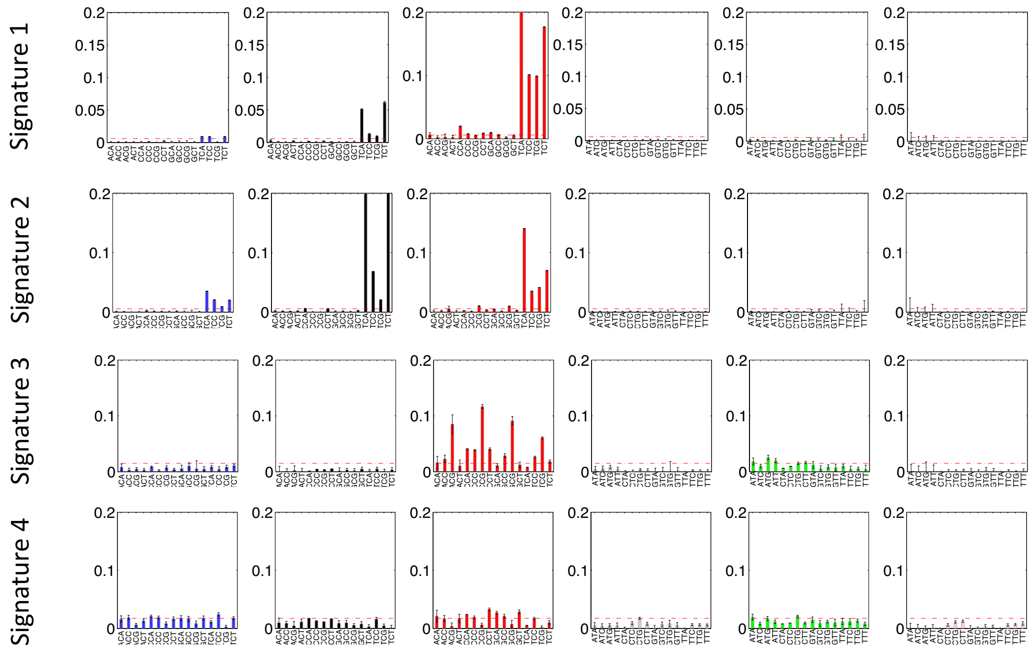

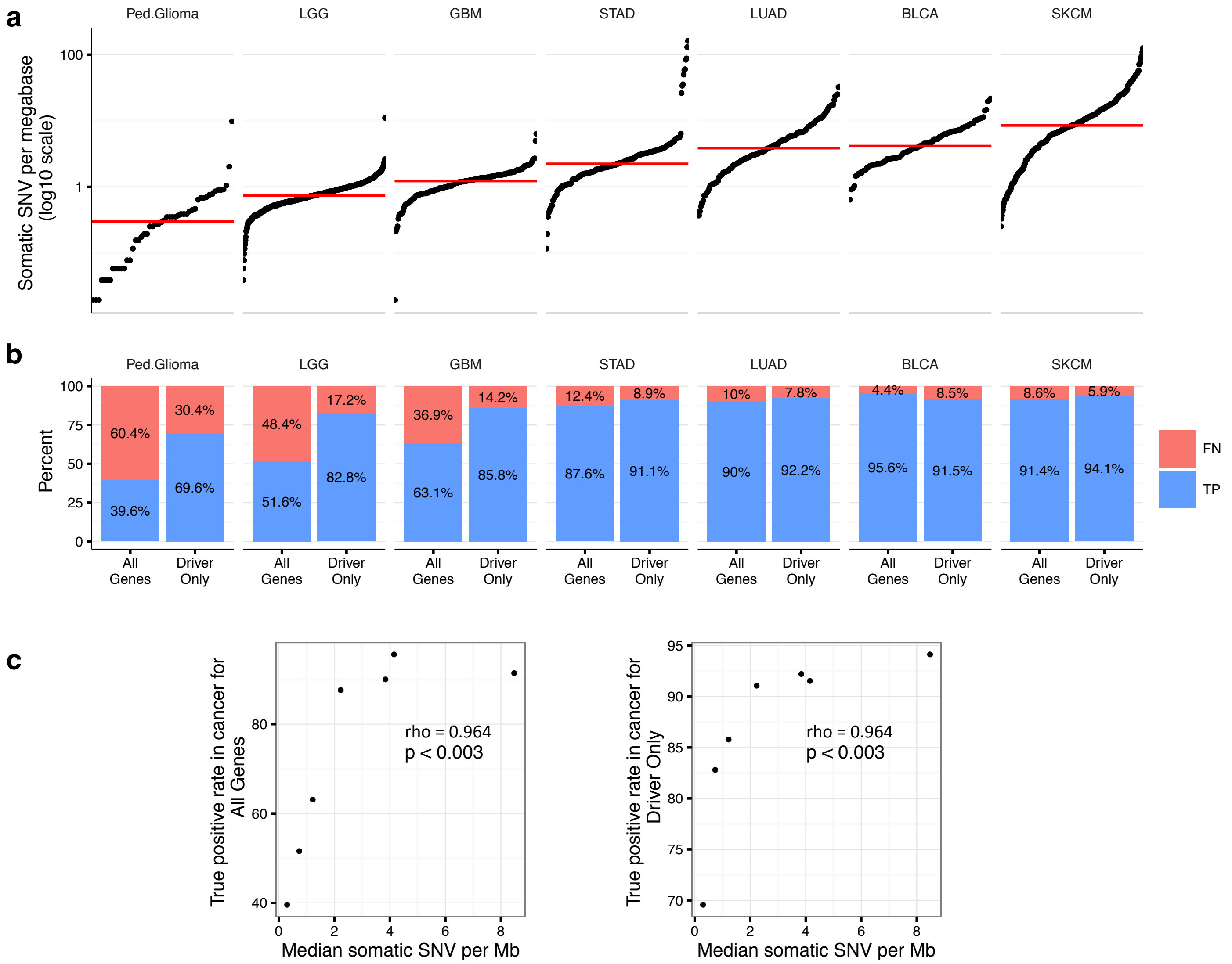

Supplementary Figure 13. Sensitivity correlates with somatic SNV rate. (a) Somatic SNV per megabase (Mb) for each cancer type. Vertical axis shows the number of somatic SNV per megabase on a log10 scale. Each point represents a tumor sample, red horizontal lines indicate median value for cancer; cancers ordered by increasing median number of somatic mutations. **(b)** Same as **Figure 2a** but cancers are ordered by increasing median number of somatic mutations. **(c)** Scatterplot of median somatic SNV per Mb versus true positive rate of nonsynonymous variants. Each point is a cancer type. Left panel uses true positive rate from all genes, right panel for driver genes only. P-value for Spearman correlation.
